# EBNA3C Augments Pim-1 Mediated Phosphorylation and Degradation of p21 to Promote B-Cell Proliferation

**DOI:** 10.1371/journal.ppat.1004304

**Published:** 2014-08-14

**Authors:** Shuvomoy Banerjee, Jie Lu, Qiliang Cai, Zhiguo Sun, Hem Chandra Jha, Erle S. Robertson

**Affiliations:** 1 Department of Microbiology and the Tumor Virology Program, Abramson Comprehensive Cancer Center, Perelman School of Medicine at the University of Pennsylvania, Philadelphia, Pennsylvania, United States of America; 2 Key Laboratory of Molecular Medical Virology (Ministries of Education and Health), School of Basic Medical Sciences, Shanghai Medical College, Fudan University, Shanghai, People's Republic of China; University of North Carolina at Chapel Hill, United States of America

## Abstract

Epstein–Barr virus (EBV), a ubiquitous human herpesvirus, can latently infect the human population. EBV is associated with several types of malignancies originating from lymphoid and epithelial cell types. EBV latent antigen 3C (EBNA3C) is essential for EBV-induced immortalization of B-cells. The Moloney murine leukemia provirus integration site (PIM-1), which encodes an oncogenic serine/threonine kinase, is linked to several cellular functions involving cell survival, proliferation, differentiation, and apoptosis. Notably, enhanced expression of Pim-1 kinase is associated with numerous hematological and non-hematological malignancies. A higher expression level of Pim-1 kinase is associated with EBV infection, suggesting a crucial role for Pim-1 in EBV-induced tumorigenesis. We now demonstrate a molecular mechanism which reveals a direct role for EBNA3C in enhancing Pim-1 expression in EBV-infected primary B-cells. We also showed that EBNA3C is physically associated with Pim-1 through its amino-terminal domain, and also forms a molecular complex in B-cells. EBNA3C can stabilize Pim-1 through abrogation of the proteasome/Ubiquitin pathway. Our results demonstrate that EBNA3C enhances Pim-1 mediated phosphorylation of p21 at the Thr^145^ residue. EBNA3C also facilitated the nuclear localization of Pim-1, and promoted EBV transformed cell proliferation by altering Pim-1 mediated regulation of the activity of the cell-cycle inhibitor p21/WAF1. Our study demonstrated that EBNA3C significantly induces Pim-1 mediated proteosomal degradation of p21. A significant reduction in cell proliferation of EBV-transformed LCLs was observed upon stable knockdown of Pim-1. This study describes a critical role for the oncoprotein Pim-1 in EBV-mediated oncogenesis, as well as provides novel insights into oncogenic kinase-targeted therapeutic intervention of EBV-associated cancers.

## Author Summary

The oncogenic serine/threonine kinase Pim-1 is upregulated in a number of human cancers including lymphomas, gastric, colorectal and prostate carcinomas. EBV nuclear antigen 3C (EBNA3C) is essential for EBV-induced transformation of human primary B-lymphocytes. Our current study revealed that EBNA3C significantly enhances Pim-1 kinase expression at both the transcript and protein levels. EBNA3C also interacts with Pim-1 and can form a complex in EBV-transformed cells. Moreover, EBNA3C increases nuclear localization of Pim-1 and stabilizes Pim-1 protein levels by inhibiting its poly-ubiquitination. Additionally, EBNA3C augments Pim-1 mediated phosphorylation of p21 and its proteosomal degradation. Stable knockdown of Pim-1 using si-RNA showed a significant decrease in proliferation of EBV transformed lymphoblastoid cell lines and subsequent induction of apoptosis by triggering the intrinsic apoptotic pathway. Therefore, our study demonstrated a new mechanism by which the oncogenic Pim-1 kinase targeted by EBV latent antigen 3C can inhibit p21 function, and is therefore a potential therapeutic target for the treatment of EBV-associated malignancies.

## Introduction

Epstein-Barr virus (EBV), a ubiquitous lymphotropic herpesvirus, latently infects human populations worldwide [1]. EBV infection is typically asymptomatic and is an important etiological factor which contributes to different human malignancies [2]. EBV is consistently associated with nasopharyngeal carcinoma (NPC) [3], African Burkitt's lymphoma (BL) [4], post-transplantation lymphoproliferative disease (PTLD) [5], Hodgkin's disease (HD) [6], and AIDS-related non-Hodgkin's lymphomas (AIDS-NHL) [7]. Additionally, EBV is also found in a fraction of gastric carcinomas particularly in Asian and African countries [8]. EBV has the potential to transform human B-lymphocytes in vitro by maintaining a continuous proliferative state, known as “immortalization” which generates permanent lymphoblastoid cell lines (LCLs) [9]. The LCLs which are produced in culture carry the viral genome as extra-chromosomal episomes and express nine latent EBV proteins including, the six nuclear antigens (EBNA 1, 2, 3A, 3B, 3C & LP), an additional three membrane associated proteins (LMP1, LMP2A & 2B), and the two EBV-encoded small RNAs (EBERs) [10]. These viral factors help to activate the quiescent B-cells from G_0_ into the cell cycle, and to sustain proliferation and maintenance of the viral genome [11].

Among the potential EBV latent antigens, EBNA3A, EBNA3B, and EBNA3C are sequentially encoded in the EBV genome and generate protein products of approximately 1,000 aa. Moreover, the EBNA3A, EBNA3B, and EBNA3C amino- terminal homologous domains are associated with RBP-Jk which mediates the association of EBNA2 and Notch with DNA [12]. EBNA3C and EBNA3A are also essential for EBV to drive primary human B-lymphocytes into continuously proliferating LCLs and for maintaining LCL growth [13]. Notably, Epstein-Barr virus nuclear antigen 3C (EBNA3C) plays an intricate regulatory role in the transcription of several viral and cellular genes [14]. EBNA3C targeted RBP-Jκ antagonizes EBNA2-mediated transactivation [15], and cooperates with EBNA2 in activating the major viral LMP1 promoter [16]. EBNA3C was found to regulate chromatin remodeling by recruiting histone acetylase and deacetylase activities [17]. Moreover, EBNA3C modulates the transcriptional level of cellular genes which are involved in cell migration and invasion by targeting the metastasis suppressor Nm23-H1 [18]. In addition, EBNA3C can modulate diverse cellular functions, presumably mediated by direct protein–protein interactions [19]. EBNA3C also stabilizes c-Myc and interacts with Mdm2 to modulate p53 mediated transcription and apoptotic activities [20], [21]. Interestingly, EBNA3C was found to be crucial for regulating the activity of cellular kinases. Recently, we have shown that EBNA3C enhances the kinase activity of cell-cycle regulatory protein Cyclin D1 which allows for subsequent ubiquitination and degradation of the tumor suppressor pRb [22].

Provirus integration site for Moloney murine leukemia virus (Pim-1), a proto-oncogene encoding a serine/threonine kinase, is linked to several cellular functions involving cell survival, proliferation, differentiation, and apoptosis [23]. It was reported that overexpression of Pim-1 is associated with the development and progression of multiple hematopoietic malignancies such as B-cell lymphomas, erythroleukemias, and acute myelogenous leukemia, T-cell lymphomas, and non-hematological malignancies including, oral squamous cell carcinoma, and prostate cancer [24]. During the process of embryo development, Pim-1 is highly expressed in liver, spleen and bone marrow in typical hematopoietic progenitors [25], [26], neonatal heart [27], central nervous system [28], and mammary gland [29]. Surprisingly, at the adult stage, Pim-1 is only slightly expressed in circulating granulocytes [26]. Previous reports also indicated that heterologous expression of Pim-1 in transgenic mice leads to increased lymphoproliferation and inhibition of apoptosis [30]. Augmented expression of Pim-1 in lymphoid cells by transgenesis highlighted its potential for oncogenesis [31]. Being a potent serine/threonine kinase, Pim-1 is able to phosphorylate itself [32], [33], through an autophosphorylation site that diverges from its consensus phosphorylation motif [34]. Several Pim-1 substrates have been identified, including p21Cip1/WAF1 [35], [36], Cdc25A [37], PTPU2 [38], NuMA [39], C-TAK1 [40], and Cdc25C [41], indicating a crucial role for Pim-1 in cell proliferation through both the G1/S and G2/M phase transition. Pim-1 also possesses anti-apoptotic activity [42], and recent reports have demonstrated a role for Pim kinases in regulation of herpesviral oncogenesis. KSHV encoded LANA was found to be crucial for transcriptional activation of Pim-1 in KSHV-positive cells and it also acts as a Pim-1 substrate [43]. In the context of EBV infection, studies have shown that Pim-1 may be required for LMP1-induced cell survival [44]. Furthermore, the expression levels of Pim-1 and Pim-2 are up-regulated upon EBV infection and they in turn enhance the activity of the viral nuclear antigen EBNA2, suggesting a role in driving EBV-induced immortalization [45]. However, the molecular mechanism by which Pim-1 is activated through expression of viral antigens which creates a micro-environment for B-cell transformation is not fully elucidated.

In our current study, we demonstrated that EBNA3C is responsible for inducing Pim-1 expression in EBV transformed B-cells as well as in EBV-infected PBMCs. Further, we showed that EBNA3C interacts with Pim-1 through a small N-terminal domain (amino acids 130–159) and forms a complex in B-cells. Our results demonstrated that EBNA3C stabilized the Pim-1 protein by inhibiting its degradation by the ubiquitin/proteasome pathway. Interestingly, EBNA3C also facilitated the nuclear localization of Pim-1, and promotes EBV-induced cell proliferation by regulating Pim-1 mediated degradation of p21/WAF1. We observed that deregulation of p21 ultimately resulted in higher cellular proliferation. Lentivirus mediated stable knockdown of Pim-1 resulted in a significant reduction of EBV transformed cells and induction of apoptosis. Cumulatively, these findings demonstrate a vital role for Pim-1 in EBV-mediated oncogenesis and also support the conclusions that Pim-1 kinase is a potential target for therapeutic intervention strategies against EBV associated malignancies.

## Results

### EBNA3C upregulates Pim-1 expression

**Figure 1:**
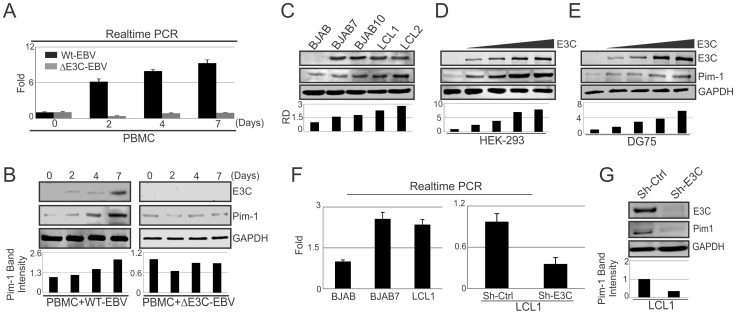
Upregulation of Pim-1 expression by EBNA3C. A) 10×10^6^ human PBMC (Peripheral blood mononuclear cells) were infected with wild type or ΔEBNA3C mutant BAC-GFP EBV for 6 hrs at 37°C. Cells were harvested after 0, 2, 4, 7, 15 days of post-infection. Total RNA was isolated and subjected to quantitative real-time PCR analysis to detect Pim-1 mRNA level. B) Wild type or ΔEBNA3C mutant BAC-GFP EBV infected cells were lysed in RIPA buffer. Western blot analysis was performed with indicated antibodies to detect specific endogenous proteins. C) 50 million EBV negative BJAB, EBNA3C expressing BJAB7, BJAB10, EBV transformed LCL1, LCL2 cells were harvested and total cell lysates were subjected to Western blot analysis (WB) using indicated antibodies. D) 10 million HEK-293 cells and E) 50 million EBV negative DG75 cells were transfected with increasing amount of EBNA3C expressing construct (0, 5, 10, 15 µg) and Western blot analysis was performed to detect Pim-1, EBNA3C, GAPDH proteins. F) Total RNA was isolated from BJAB, BJAB7, LCL1, sh-Ctrl LCL1, sh-E3C LCL1 cells and subjected to quantitative real-time PCR analysis to detect *Pim-1* mRNA levels. G) Lentivirus mediated stable EBNA3C knockdown (sh-E3C) or scramble control (sh-Ctrl) LCL1 cells were subjected to Western blot analysis with indicated antibodies. Protein bands from Western blot analysis were analyzed by the Odyssey imager software and represented as bar diagrams based on internal loading control GAPDH.

Pim-1 expression was found upregulated in different hematological and non-hematological malignancies [23]. To determine whether EBV latent antigen 3C modulates Pim-1 expression, 10 million human peripheral blood mononuclear cells (PBMC) were infected by wild type and mutant ΔEBNA3C BAC-GFP-EBV for 4 hrs at 37°C described previously [46]. The mRNA and protein levels of Pim-1 were detected after 0, 2, 4, 7 days of infection. Our results showed upregulation of both the transcript and protein levels of Pim-1 with wild type EBV infection (Fig. 1A). Interestingly, infection with ΔEBNA3C BAC-GFP-EBV resulted in low Pim-1 expression at 2 days post-infection and returned to the levels seen for infected cells at 0 day post-infection (Fig. 1B). The results indicated that Pim-1 expression was induced by wild-type EBV infection. Therefore, we wanted to determine the expression pattern of Pim-1 in EBV transformed Lymphoblastoid cells LCL1, LCL2, and EBNA3C stably expressing BJAB7 and BJAB10 cells when compared to EBV negative BJAB. Our results showed that Pim-1 expression was highly upregulated in LCL1, LCL2, BJAB7 and BJAB10 cells (Fig. 1C). To investigate the role of EBNA3C on Pim-1, we monitored the Pim-1 protein expression levels with a dose dependent increase of EBNA3C in EBV negative DG75 as well as in HEK-293 cells. The results showed a steady increase in Pim-1 expression levels in both cell lines (Fig. 1D and 1E). Moreover, Real-time PCR analysis showed upregulation of *Pim-1* mRNA expression in BJAB7 and LCL1 cells when compared to EBV negative BJAB cells (Fig. 1F, left panel). To further investigate the role of EBNA3C in inducing Pim-1 expression, we performed Real-time PCR as well as Western blot analysis on EBNA3C stable knock-down LCL1 cells. The results demonstrated a substantial reduction of Pim-1 expression in both mRNA and protein levels as compared with sh-control LCL1 cells (Fig. 1F, right panel and Fig. 1G). Moreover, to check the role of other EBV antigens including EBNA2, EBNA3A, and EBNA3B on *Pim-1* expression, we performed si-RNA mediated knockdown of EBNA2, EBNA3A, EBNA3B and EBNA3C in LCL1 cells. Our Real-time PCR analysis demonstrated that *Pim-1* mRNA level is significantly reduced upon EBNA3C knockdown but no significant change was observed in *Pim-1* mRNA expression level with EBNA2, EBNA3A, EBNA3B knockdown further suggesting a major role for EBNA3C in upregulating *Pim-1* expression (Fig. S1A and S1B). Additionally, we performed Western blot analysis to determine whether knock down of EBNA3C may have an effect on other EBNAs expression levels. The results demonstrated that expression levels of other EBNAs were not affected with EBNA3C knockdown (Fig. S2).

### EBNA3C associates with Pim-1 in human B-cell lines

**Figure 2:**
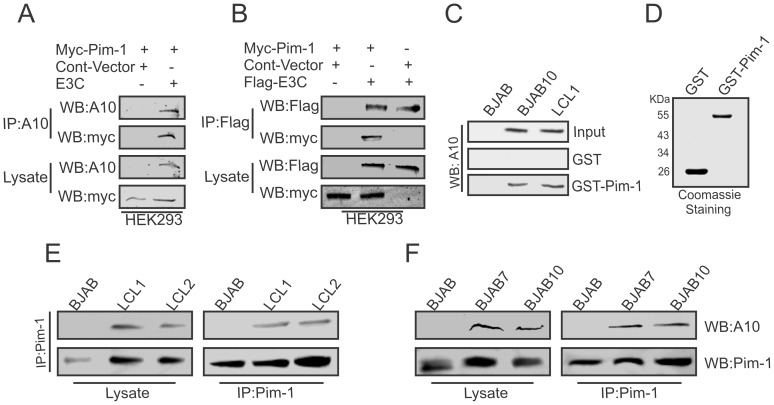
EBNA3C physically associates with Pim-1. A–B) 10 million HEK- 293 cells were co-transfected with Myc-tagged Pim-1, untagged-EBNA3C or Flag-tagged EBNA3C expression vectors. Control samples were balanced by using empty vector. Transfected cells were harvested at 36 hrs of post-transfection and approximately 5% of the lysates were used as input and the residual lysate was immunoprecipitated (IP) with 1 µg of anti-Flag (M2) or A10 antibodies. Lysates and Immunoprecipitated samples were resolved by 10% SDS-PAGE and western blot (WB) analysis was performed with the indicated antibodies. C) 50 million BJAB, BJAB7, LCL1, were harvested and lysed in RIPA buffer. Cell lysates were incubated with either GST control or GST-Pim-1 beads. EBNA3C protein was detected by western blot analysis using EBNA3C specific monoclonal antibody (A10). D) Purified control GST and GST-Pim-1 proteins used in this experiment were resolved by 10% SDS-PAGE and stained with Coomassie Blue. 50 million E–F) BJAB, BJAB7, BJAB10, LCL1, and LCL2 cells were lysed and immunoprecipitation was performed by Pim-1 specific antibody. Immunoprecipitated samples were resolved by 10% SDS-PAGE and endogenous EBNA3C, Pim-1 proteins were detected by their specific antibodies.

To determine whether EBNA3C interacted with Pim-1, we performed co-immunoprecipitation experiments in HEK-293 cells by expressing Myc-tagged Pim-1, Flag-EBNA3C, or Myc-EBNA3C. Immunoprecipitation was performed using A10 (Fig. 2A) or 9E10 antibody (Fig. 2B). The results clearly demonstrated that EBNA3C strongly associated with Pim-1 (Fig. 2A and B). We further supported our results by GST-pull down assays using EBV negative BJAB, EBNA3C expressing BJAB10 and EBV transformed LCL1 cell lysates incubated with bacterially purified GST-Pim-1 protein. EBNA3C was detected by A10 antibody [47] which showed a substantial level of association between Pim-1 and EBNA3C in the EBNA3C stable cell lines as well as in an LCL (Fig. 2C). Coomassie staining of bacterially purified GST and GST-Pim-1 proteins are shown in Fig. 2D. We also observed the association between EBNA3C and Pim-1 in BJAB7, BJAB10, LCL1, LCL2 cells compared with BJAB in separate co-immunoprecipitation experiments by using Pim-1 specific antibody (Fig. 2E and 2F).

### Pim-1 specifically binds to the N-terminal domain of EBNA3C

**Figure 3:**
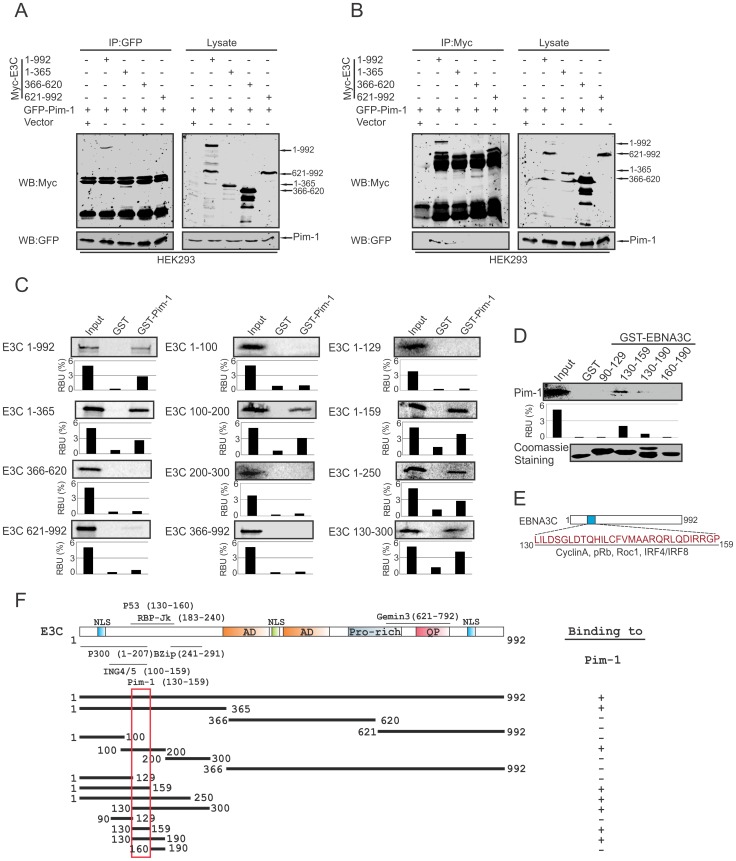
Binding of Pim-1 to the N-terminal EBNA3C domain. A–B) 10 million HEK-293 cells were transfected with either control vector or Full length and different truncated mutants of Myc-tagged EBNA3C with GFP-tagged Pim-1 plasmid constructs. After 36 hours of post-transfection, cells were harvested and immunoprecipitation performed with 1 µg of GFP or anti-Myc antibodies. IP samples were resolved in 10% SDS-PAGE. Western blot was performed with anti-Myc and anti-GFP antibodies. C) Full length and different domains truncated mutant constructs of EBNA3C were in vitro translated using a T7-TNT translation kit. After pre-clearing with GST-beads, all S^35^-radiolabeled in vitro translated proteins incubated with either GST control or GST-Pim-1 beads. Reaction samples were washed with Binding Buffer and resolved by 10% SDS-PAGE, exposed to phosphoimager plate and scanned by Typhoon Scanner. D) Myc-Pim-1 construct were used for in vitro translation and S^35^-radiolabeled in vitro translated proteins were incubated with either GST control or different GST-EBNA3C truncated mutant beads. Coomassie staining of SDS-PAGE resolved purified GST proteins is shown in the bottom panel of D). In each case, 5% of IVT input was used for the comparison. E) The diagram shows EBNA3C 130–159 amino acids motif important binding sites for different cellular proteins. F) The schematic diagram represents various structural and interactive domains of EBNA3C and summarizes the binding affinities between different domains of EBNA3C with Pim-1. +, binding; −, no binding.

To determine the specific domain of EBNA3C associated with Pim-1, we performed co-immunoprecipitation experiments expressing GFP-tagged Pim-1 with Myc-tagged full length (residues 1–992) and different truncated mutants (residues 1–365, 366–620 and 621–992) of EBNA3C in HEK-293 cells. Immunoprecipitation (IP) was performed by using either 9E10 or GFP-specific antibodies. The results indicated that Pim-1 strongly associated with full length as well as the N-terminal domain (residues 1–365) of EBNA3C (Fig. 3A and 3B). We extended the binding experiments by performing *in vitro* GST-pulldown assay with in vitro translated full length and truncated mutants of EBNA3C including fragments within the N-terminal domain (residues 1–992, 1–365, 366–620, 621–992, 1–100, 100–200, 200–300, 366–992, 1–129, 1–159, 1–250, 130–300). Our results demonstrated that EBNA3C residues 100–200, 1–159, 1–250, 130–300 associated strongly with full length Pim-1 (Fig. 3C). To further map the specific binding residues, we performed additional *in vitro* GST-pulldown assays by using *in vitro* translated full length Pim-1 incubated with bacterially expressed N-terminal truncated mutants of GST-EBNA3C fused to residues 90–129, 130–159, 130–190, 160–190. The results indicated that Pim-1 strongly bound to residues 130–159 of EBNA3C (Fig. 3D, 3E and 3F).

### EBNA3C facilitates nuclear transport and co-localizes with Pim-1

**Figure 4:**
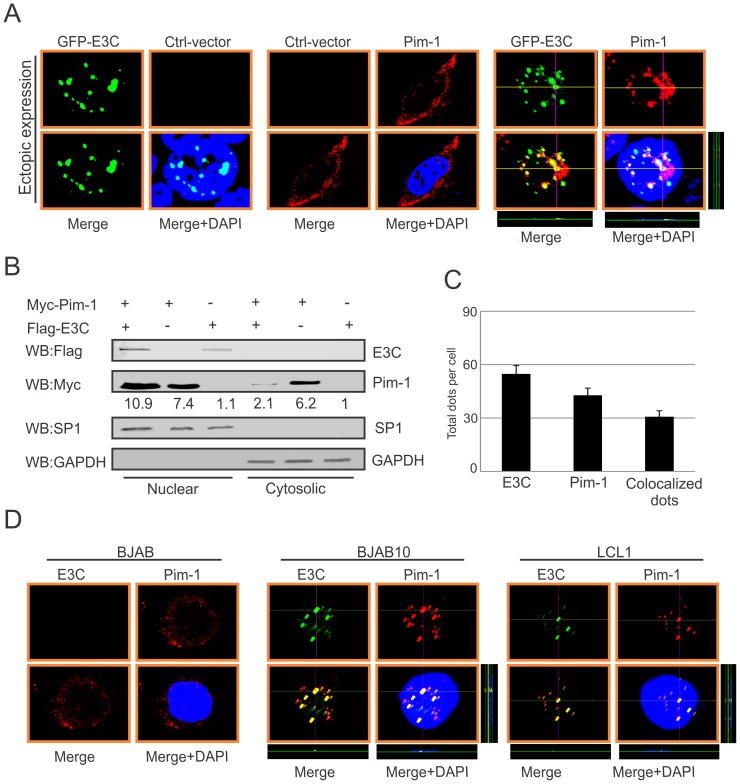
EBNA3C co-localizes with Pim-1 and facilitates its nuclear translocation. A) 0.3 million HEK-293 cells plated on coverslips and transiently transfected with control vector, GFP-EBNA3C and Myc-Pim-1 expression vectors by using Lipofectamine 2000 transfection reagent. B) 10 million HEK-293 cells were transfected with Myc-Pim-1 and Flag-EBNA3C and subjected to sub-cellular fractionation assay. C) Bar diagram represents quantitation of co-localization in panel A. D) BJAB, BJAB10, LCL1 cells were plated on slides and air-dried. Ectopic and endogenous expressions of Pim-1 was detected using anti-Myc (9E10)-antibody (1∶200 dilution) and Pim-1 specific antibody (1∶50 dilution) respectively, followed by anti-Rabbit Alexa Fluor 594 and anti-goat Alexa Fluor 555 (red) as secondary antibodies. Endogenous EBNA3C was detected using A10 ascites (1∶150 dilution) followed by anti-mouse Alexa Fluor 488 (green). DAPI (49, 69-diamidino-2-phenylindole) was used (1∶500 dilution) to stain nuclei. The images were captured by Olympus Fluoview confocal microscope.

In the context of cancer progression, the significance of different subcellular localization patterns of Pim-1 has not been fully elucidated. Previous studies suggested that irradiation can promote nuclear translocation of Pim-1 in radio-resistant squamocellular malignancies of head and neck [48]. Importantly, nuclear localization of Pim-1 may correlate with the proliferating cells and may also contribute to a survival response upon pathologic injury [49]. In our study, we transfected Myc-tagged Pim-1 with or without GFP-tagged EBNA3C expression vectors in HEK-293 cells. Cellular localization of Pim-1 was examined by immunofluorescence analysis using specific antibodies against the Myc-epitope. Interestingly, our results showed that the localization of Pim-1 was predominantly in the cytoplasm without EBNA3C and was translocated to the nucleus in the presence of EBNA3C. Also, strong co-localization with Pim-1 and EBNA3C was observed (Fig. 4A and 4C). To further validate these results, we performed nuclear and cytosolic fractionation assays using transiently transfected HEK-293 cells with Myc-tagged Pim-1 with or without Flag-tagged EBNA3C expression vectors. Our Western blot analysis with nuclear and cytosolic fractions showed that in the presence of EBNA3C, the level of Pim-1 substantially increased in the nuclear fraction (Fig. 4B). Moreover, we corroborated the above observations in EBV negative BJAB, EBNA3C stably expressing BJAB10 and EBV transformed LCL1 cells using specific antibodies against Pim-1 and EBNA3C. The results showed that Pim-1 was mostly localized in the nucleus in both EBNA3C expressing BJAB10 and EBV transformed LCL1 cells, but was almost entirely cytoplasmic in EBV negative BJAB cells (Fig. 4D).

### EBNA3C enhances the stability of Pim-1 in EBV-transformed LCLs

**Figure 5:**
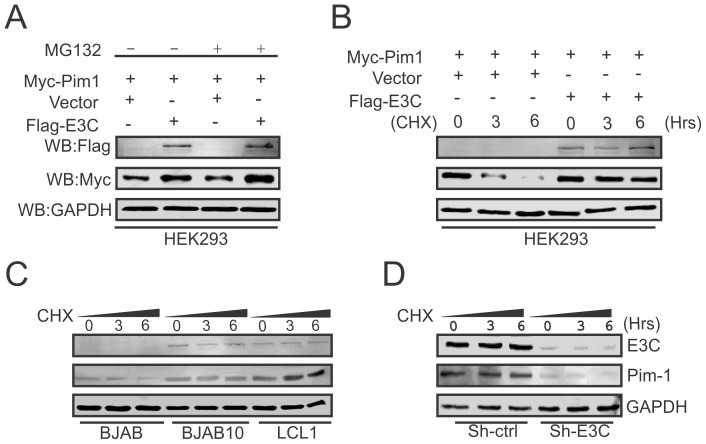
EBNA3C contributes to Pim-1 stabilization. A) 10 million HEK-293 cells were co-transfected with Myc-Pim-1 and either vector control (lanes 1 and 3) or Flag-EBNA3C (lanes 2 and 4) expression constructs. After 36 hrs of post-transfection, transfected cells were treated with either 20 µM MG132 (+ lanes) or DMSO (− lanes) for additional 6 hrs and cell lysates were resolved by 10% SDS-PAGE and Western blot was performed with the indicated antibodies. B) HEK-293 cells were transfected with above mentioned expression vectors and At 36 hrs of post-transfection, cells were treated with 40 µg/ml cyclohexamide (CHX) for 0, 3, 6 hrs. Cells were lysed and protein samples were resolved by 10% SDS-PAGE. Western blot was performed by specific antibodies shown. C–D) BJAB, BJAB10, LCL1, sh-Ctrl and sh-EBNA3C cells were treated with 40 µg/ml cyclohexamide (CHX) for indicated time periods. Cell lysates were resolved by 10% SDS-PAGE. Western blot analysis was performed with indicated antibodies. GAPDH blot was shown for internal loading control.

Recent reports suggested that expression of EBNA3C is responsible for the stabilization of different oncoproteins, transcription factors and cellular kinases [19], [22], [50], [51], and also plays an important role in modulating the ubiquitin (Ub)-proteasome machinery [52]. Our results so far showed that EBNA3C is important for enhanced protein expression of Pim-1. To determine, if this induced expression is related to EBNA3C-mediated stabilization of Pim-1 by the inhibition of Ub-proteosome machinery, we co-transfected Myc-tagged Pim-1 with or without Flag-tagged EBNA3C expression plasmids in HEK-293 cells which were treated with or without the proteasome inhibitor MG132. The results showed a substantial accumulation of Pim-1 protein levels in MG132 treated cells in the presence of EBNA3C compared with mock treatment and control vector (Fig. 5A). Next, we performed the stability assay of Pim-1 by transfecting Myc-tagged Pim-1 with or without Flag-tagged EBNA3C in HEK-293 cells. After 36 hours of post-transfection, cells were treated with the protein synthesis inhibitor cyclohexamide and harvested at 0, 3, and 6 hours intervals. The Western blot results clearly demonstrated that Pim-1 levels were stabilized with co-expression of EBNA3C whereas, the Pim-1 expression levels were markedly reduced with cyclohexamide treatment by 3 to 6 hours in the absence of EBNA3C (Fig. 5B). To further corroborate our results, we extended the stability assays with EBV negative BJAB, EBNA3C stably expressing BJAB10 and EBV transformed LCL1, control vector transfected and EBNA3C stably knockdown LCL1 cells. As anticipated, our results showed that Pim-1 protein levels were stabilized in BJAB10, LCL1 and sh-Ctrl LCL1 cells as well but significantly reduced in BJAB, sh-E3C LCL1 cells over time with the treatment of cyclohexamide (Fig. 5C and 5D).

### EBNA3C can inhibit poly-ubiquitination of Pim-1

**Figure 6:**
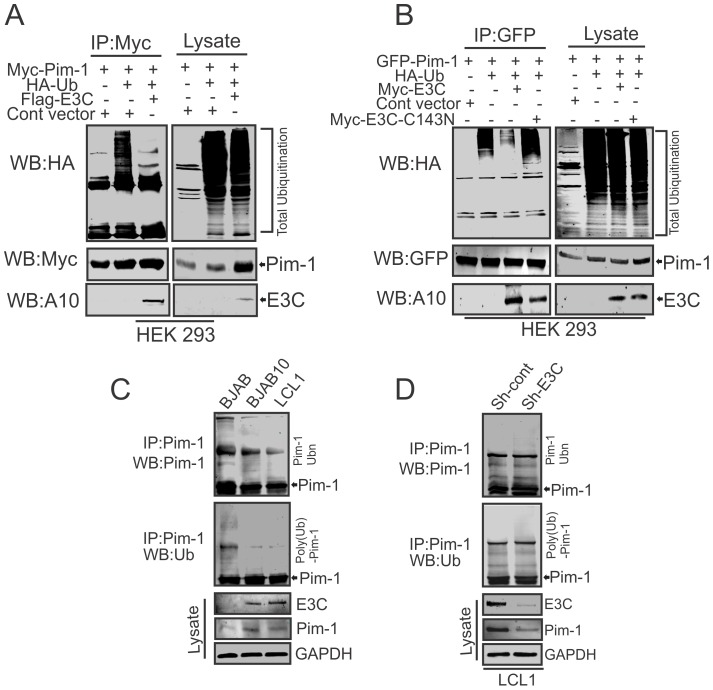
EBNA3C suppresses poly-ubiquitination of Pim-1. A–B) 10 million HEK-293 cells were transiently transfected with different combinations of expression vectors as indicated. Cells were harvested after 36 hrs of post-transfection by incubating with proteasome inhibitor MG132 drug for additional 6 hrs. Total protein was immunoprecipitated (IP) with indicated antibodies and protein samples were resolved by 10% SDS-PAGE. Western blots were performed by stripping and re-probing the same membrane. C–D) 50 million EBV negative BJAB cells, BJAB10, LCL1, sh-Ctrl, sh-Pim-1 LCL1 cells were incubated with proteasome inhibitor MG132 drug (20 µM) for 6 hrs. Cells were harvested and lysed with RIPA buffer. IRF4 was immunoprecipitated (IP) by using specific antibodies. Samples were resolved by 10% SDS-PAGE. Western blotting (WB) was performed by stripping and re-probing the same membrane.

The enhanced stability of Pim-1 in the presence of EBNA3C encouraged us to investigate the role of EBNA3C for regulating Pim-1 poly-ubiquitination. Therefore, we performed *in vivo* poly-ubiquitination assays in cells by co-transfecting with control vector, Myc-tagged Pim-1, HA-Ubiquitin, with or without Flag-tagged EBNA3C in HEK-293 cells. The results demonstrated a significant reduction of Pim-1 poly-ubiquitination levels in the presence of EBNA3C (Fig. 6A). To further validate the role of EBNA3C, we performed poly-ubiquitination assays by using the wild type Myc-tagged EBNA3C and its specific mutant EBNA3C (Myc-EBNA3C-C143N) expression vectors. We observed higher poly-ubiquitination levels of Pim-1 in the presence of the EBNA3C-C143N mutant compared with wild type (Fig. 6B). We also performed the ubiquitination assays in a B-cell background by using EBV-negative BJAB, EBNA3C stably expressing BJAB10 and EBV transformed lymphoblastoid LCL1, as well as the sh-Ctrl and sh-EBNA3C LCL1 cell lines. Our result showed that the status of Pim-1 ubiquitination was much lower in BJAB10 and LCL1 cells compared with BJAB (Fig. 6C) and somewhat enhanced upon EBNA3C knockdown (Fig. 6D).

### EBNA3C enhances Pim-1 kinase-mediated phosphorylation of the Cyclin inhibitor p21 at the threonine 145 residue

**Figure 7:**
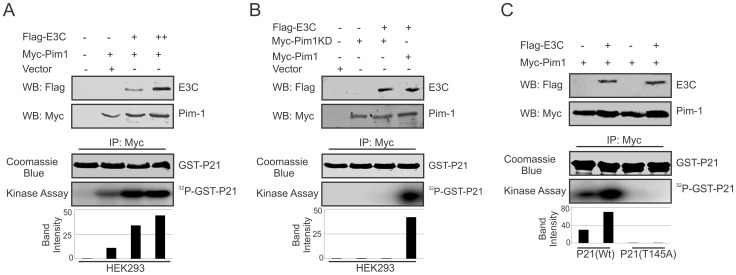
EBNA3C enhances Pim-1 kinase mediated phosphorylation of p21 Thr^145^ residue. A–C) HEK-293 cells were transfected with Myc-tagged Pim-1 (wild type or kinase dead mutant) and Flag-tagged EBNA3C vectors as indicated. Empty vector was used to balance total transfected DNA. At 36 h post-transfection, Pim-1 immunoprecipitates were captured with anti-Myc antibody and assayed for in vitro kinase activity toward GST-p21 (wild type or T145A mutant) using γP ^32^-ATP. Western blot using whole cell lysates and Coomassie staining of SDS-PAGE resolved GST proteins used in this study is shown here.

The serine/threonine-protein kinase Pim-1 is upregulated in a number of hematological malignancies such as leukemia [26], mantle-cell lymphoma [53], and diffuse large B-cell lymphoma (DLBCL) [54]. A wide range of Pim-1 substrates were identified including, BAD [55], NuMa [39], Socs [56], Cdc25A [37], C-TAK1 [40], NFATc [57], HP-1 [58], PAP-1 [59], and cyclin-dependent kinase inhibitor p21 or p21Cip1/WAF1 [35], which suggested that Pim-1 can function at different cellular events, such as cell proliferation, differentiation, and cell survival [60]. Earlier reports showed that p21 suppresses tumors by promoting cell cycle arrest in response to various stimuli. Furthermore, considerable evidence from biochemical and genetic studies have demonstrated that p21 can act as a master effector molecule of multiple tumor suppressor pathways for promoting anti-proliferative activities which are independent of classical p53 tumor suppressor pathway [61]. Studies have also shown that enhanced levels of Pim-1 kinase phosphorylates Thr^145^ residue, and regulates the activity of p21Cip1/WAF1 [35]. Therefore, we checked the kinase activity of Pim-1 towards its substrate p21 with or without EBNA3C to investigate whether EBNA3C can modulate the phosphorylation status of p21. HEK-293 cells were transiently transfected with control vector, with and without Myc-tagged Pim-1 and increasing doses of Flag-tagged EBNA3C expression vectors. Immunoprecipitation was performed using anti-Myc 9E10 antibody and immunoprecipitated complexes were further examined for in vitro kinase activity as determined by GST-p21 phosphorylation. Interestingly, the results demonstrated that the ability of Pim-1 kinase to phosphorylate p21 was substantially and proportionally augmented by a dose-dependent increase in EBNA3C expression (Fig. 7A). We further extended the kinase assay using a kinase-dead (KD) mutant of Pim-1 (Fig. 7B). As anticipated, there was no kinase activity observed with the kinase-dead (KD) mutant of Pim-1 when compared with wild type. Next, we performed *in vitro* kinase assay for Pim-1 in the presence or absence of EBNA3C by using wild type and mutant (T145A) GST-p21 as substrate. The results showed no phosphorylation with mutant (T145A) p21 in comparison with wild-type, even in the presence of EBNA3C (Fig. 7C). This suggested that the Thr^145^ residue is important for EBNA3C mediated enhancement of p21 phosphorylation by Pim-1 kinase.

### EBNA3C competes with p21 to inhibit the complex formation with Pim-1 kinase

**Figure 8:**
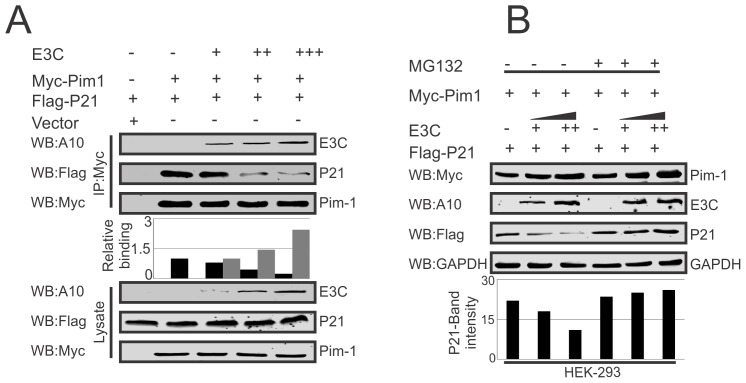
EBNA3C competes with p21 for Pim-1 binding. A–B) 10 million HEK-293 cells were transfected with different combinations of Myc-tagged Pim-1, Flag-tagged p21, untagged-EBNA3C expression vectors as indicated by electroporation. A) IP was performed with anti-Myc antibody and IP complexes were resolved by 10% SDS-PAGE. Western blot was performed with indicated antibodies. B) Above mentioned transfected cells were treated with MG132 drug for 6 hrs and protein lysates were prepared by RIPA buffer. Western blot analysis was performed by indicated antibodies.

Earlier reports suggested the potential of a complex containing Pim-1 and p21 in cells [36]. We have now confirmed a strong association between Pim-1 and EBNA3C above. We then performed competitive binding assays in HEK-293 cells by co-transfecting increasing doses of EBNA3C-expression construct and a constant amount of Myc-tagged Pim-1 and Flag-tagged p21. Immunoprecipitation (IP) was performed with anti-Myc antibody for immunoprecipitation of complex with Pim-1. Our results demonstrated that increasing doses of EBNA3C can result in reduced association between Pim-1 and p21 (Fig. 8A). Previous studies showed that p21 is a prime target for ubiquitination in gliomas [62], and was dependent on the ubiquitin ligase APC/C^Cdc20^ for its proteolytic degradation by the proteasome [63]. To explore the modulation of p21 protein levels by EBNA3C through regulation of the Ub-proteasome machinery, HEK-293 cells were co-transfected with Myc-Pim-1, Flag-p21, and increasing amounts of untagged-EBNA3C then treated with the proteasome inhibitor, MG132. The results indicated that the level of p21 was significantly reduced in the mock treated cells. However, with MG132 drug treatment, the level of p21 was further enhanced in the presence of EBNA3C (Fig. 8B).

### EBNA3C destabilizes p21 through Pim-1 kinase independent of etoposide induced DNA damage response

**Figure 9:**
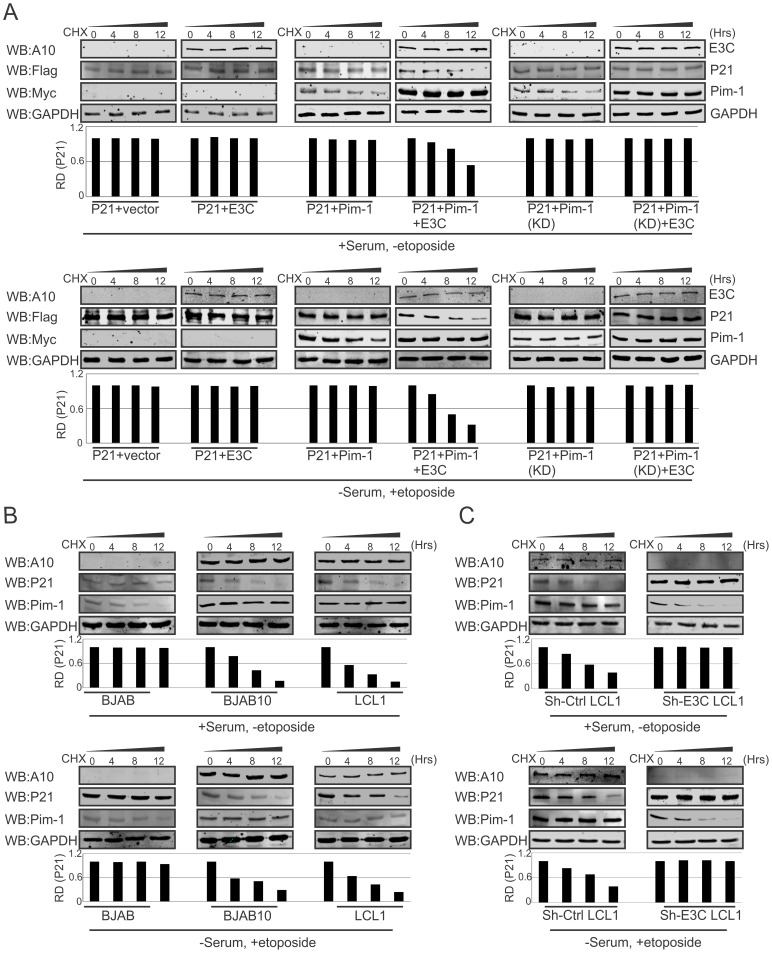
EBNA3C destabilizes p21 through Pim-1 independent of the DNA damage response. A) 10 million HEK-293 cells were transfected with combinations of Myc-tagged Pim-1, Flag-tagged p21, untagged-EBNA3C expression by electroporation. After 36 hrs of post-transfection, cells were treated with cyclohexamide for indicated time points in DMEM medium containing either serum/DMSO or 0.1% FBS with 5 µM etoposide. Protein samples were resolved by 10% SDS-PAGE and Western blots were performed with indicated antibodies. B–C) 50 million EBV negative BJAB cells, EBNA3C expressing BJAB10, EBV transformed LCL1, sh-Ctrl, sh-E3C LCL1 cells were incubated with cyclohexamide for specific time points in RPMI medium containing serum/DMSO or 0.1% FBS with 5 µM etoposide. Samples were resolved by 10% SDS-PAGE and Western blot analysis was performed with specific antibodies.

Previous reports suggested that p21 regulates fundamental cellular processes, including cell cycle progression, apoptosis, and transcription on DNA damage response [64], [65]. Interestingly, involvement of p21 in all these major signaling pathways may occur not only after DNA damage response, but also depends on physiological conditions [66], [67]. To determine whether EBNA3C alone or an EBNA3C/Pim-1 complex had a role in p21 stabilization in DNA damage response, we performed stability assays using cyclohexamide treated HEK-293 cells co-transfected with different combinations of untagged-EBNA3C, Myc-Pim-1, Myc-Pim-1 KD (kinase dead) mutant, and Flag-p21 expression constructs. The experiments were performed with or without DNA damage response signal (reduction of serum with etoposide drug treatment). Interestingly, the results demonstrated that p21 expression levels were substantially reduced with co-expression of wild type Pim-1 and EBNA3C. However, p21 expression levels remained unchanged with EBNA3C, wild type Pim-1 or kinase dead Pim-1 alone with or without DNA damage (Fig. 9A, upper and lower panels). Therefore, EBNA3C contributes to the process of p21 degradation in co-operation with wild type Pim-1. We also extended our stability assays in EBV negative BJAB, EBNA3C stably expressing BJAB10, EBV transformed lymphoblastoid LCL1, sh-Ctrl and sh-EBNA3C LCL1 cells with cyclohexamide treatment in the presence or absence of etoposide induced DNA damage. P21 protein expression was found significantly reduced in LCL1, BJAB10 compared with BJAB even both with or without DNA damage response (Fig. 9B, upper and lower panels). Importantly, the expression levels were found augmented with or without DNA damage in EBNA3C stable knockdown LCL1 cells (Fig. 9C, upper and lower panels) suggesting a role for EBNA3C in deregulating p21 stability independent of etoposide induced DNA damage response.

### EBNA3C enhances Pim-1 dependent proteasome degradation of P21

**Figure 10:**
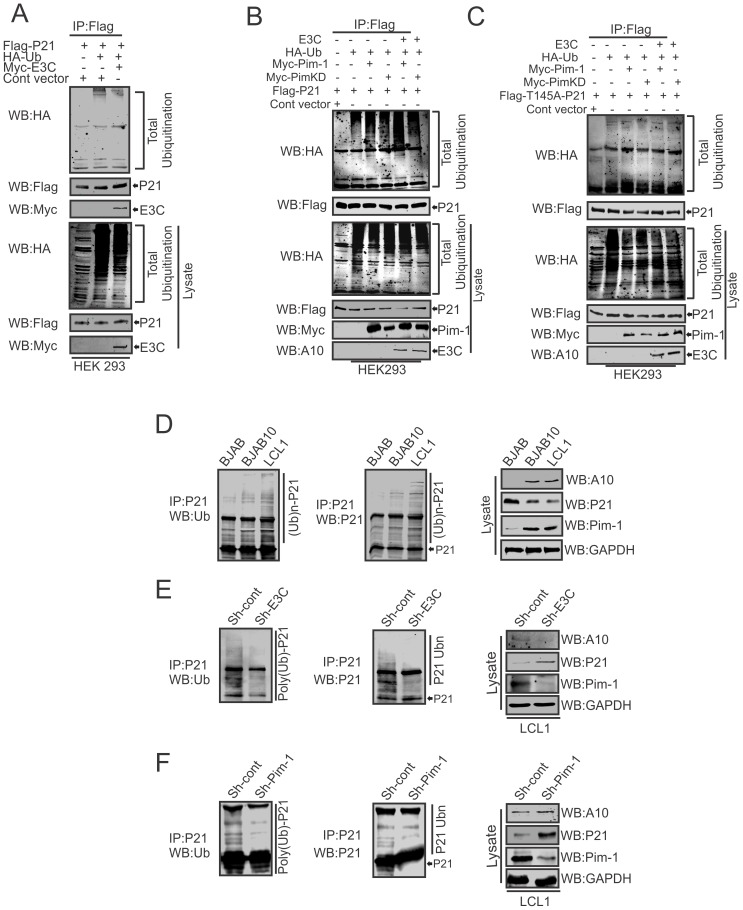
EBNA3C augments Pim-1 dependent degradation of p21. A–C) 10 million HEK-293 cells were transiently transfected with different combinations of expression vectors as indicated. Cells were harvested after 36 hrs of post-transfection by incubating with MG132 drug for additional 6 hrs and total protein was immunoprecipitated (IP) with indicated antibodies and protein samples were resolved by 10% SDS-PAGE. Western blots were performed by stripping and re-probing the same membrane. D–F) 50 million EBV negative BJAB, BJAB cells stably expressing BJAB10 and EBV transformed LCL1, sh-Ctrl, sh-E3C, sh-Pim-1 LCL1 cells were incubated with proteasome inhibitor MG132 drug (20 µM) for 6 hrs. Treated cells were harvested and lysed with RIPA buffer. p21 was immunoprecipitated (IP) by using specific antibody. Samples were resolved by 10% SDS-PAGE. Western blotting (WB) was performed by stripping and re-probing the same membrane.

To examine, whether EBNA3C has a vital role in p21 degradation alone or in collaboration with Pim-1, we performed poly-ubiquitination assays by expressing Flag-p21 and Myc-tagged EBNA3C in HEK-293 cells. The results demonstrated there was no significant change in the level of poly-ubiquitination (Fig. 10A). Our study also revealed a strong association with p21 and EBNA3C by co-immunoprecipitation experiments (Fig. S3A). Next, we attempted to examine the potential changes in p21 protein levels by expressing Flag-p21, Myc-Pim-1, along with increasing amounts of EBNA3C in HEK-293 cells. Interestingly, we observed reduced levels of p21 with a dose dependent increase of EBNA3C in the presence of Pim-1 (Fig. S3B). Our poly-ubiquitination assay results for p21, with wild-type Pim-1 and kinase-dead Pim-1 mutant clearly showed that the level of poly-ubiquitination was much higher with wild-type Pim-1 compared with kinase-dead mutant in the presence of EBNA3C (Fig. 10B). This supported an important role for EBNA3C in enhancing Pim-1 kinase activity and is likely to be required for p21 degradation. Moreover, we extended the poly-ubiquitination assays using the P21T145A mutant to determine whether the p21 Thr145 phosphorylation was related to its degradation. The results showed that levels of poly-ubiquitination remained unchanged both with wild-type and the kinase-dead mutant of Pim-1 in the presence of EBNA3C (Fig. 10C). Additionally, we performed poly-ubiquitination assay using EBV negative BJAB, EBNA3C stably expressing BJAB10, EBV transformed lymphoblastoid LCL1, as well as sh-Ctrl and sh-EBNA3C LCL1 cells to monitor the poly-ubiquitination status of p21. The results clearly indicated higher poly-ubiquitinated levels of p21 in EBNA3C expressing BJAB10, and LCL1 cells compared with EBNA3C negative BJAB (Fig. 10D). The levels were also reduced in EBNA3C stable knockdown LCL1 cells (Fig. 10E). To determine if the degradation of p21 is Pim-1 dependent, we performed poly-ubiquitination assays with Pim-1 stable knockdown LCL1 cells. The results indicated that upon Pim-1 knockdown, the level of p21 degradation was reduced (Fig. 10F).

### Pim-1 knockdown sensitizes EBV transformed cells towards induction of the cellular intrinsic apoptosis signaling pathway

**Figure 11:**
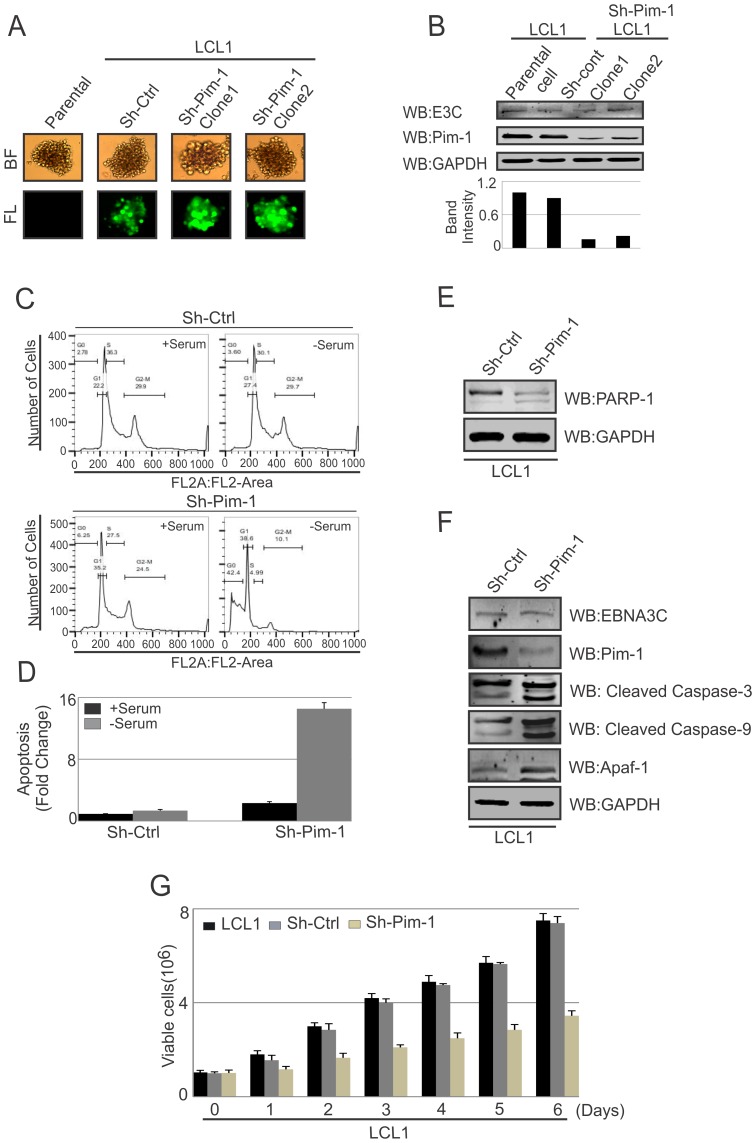
Pim-1 silencing by sh-RNA sensitizes EBV transformed lymphoblastoid cells by induction of the intrinsic apoptosis signaling pathway. A) Wild type LCL1, Lentivirus mediated and puromycin selected stable sh-Ctrl vector transfected and different clones of Pim-1 knocked down cells were observed by fluorescence microscope for monitoring GFP fluorescence B) 50 million different clones of stable sh-Pim-1, sh-Ctrl LCL1, and LCL1 cells were harvested and cell lysates were prepared by RIPA buffer. Western blot analysis was performed to show the expression levels of EBNA3C, Pim-1 and GAPDH. C) sh-Ctrl, sh-Pim-1 LCL1 cells were grown in RPMI medium for 12 hrs with or without serum starvation. Cells were stained with Propidium iodide and subjected to Flowcytometric analysis. D) Bar diagram represents the fold change of apoptosis observed by apoptosis assay using FACS. The results represented here are representative of three independent experiments. E–F) 50 million sh-Ctrl and sh-Pim-1 LCL1 cells were harvested and cell lysates were used for Western blot analysis with indicated antibodies. G) 1×10^6^ cells were plated and allowed them to grow at 37°C in complete medium without puromycin antibiotic. Viable cells were counted by trypan blue dye exclusion technique at indicated time points. The results shown here are representative of three independent experiments.

Earlier reports demonstrated that Pim-1 kinase activity is linked to enhanced cellular proliferation in neoplastic cell types [68]. To determine the effect of EBNA3C on Pim-1 mediated cell proliferation, HEK-293 cells were transfected with control vector, Flag-tagged EBNA3C, Myc-Pim-1 expression vector, and Myc-Pim-1 with Flag-EBNA3C. Colony formation assays were performed after G418 selection for 2 weeks. The results demonstrated a significant increase in the colony numbers in EBNA3C and the Pim-1 co-transfected set compared with control vector or only Pim-1 transfected sets (Fig. S4A and S4B). Additionally, cell proliferation assays were performed by cell counting using Trypan blue dye exclusion technique up to 6 days (Fig. S4C). Previous studies suggested that Pim-1 expression accelerated the process of lymphoproliferation and inhibits apoptosis [30]. Also, depletion of Pim-1 by RNA interference in mouse and human prostate cancer cells reduced cellular proliferation and survival [69]. To validate these studies, we used Lentivirus mediated delivery of sh-RNA vectors to knock down Pim-1 in LCL1 cells. Wild type LCL1, puromycin selected stable Ctrl-vector and Pim-1 knocked down cells with GFP fluorescence were monitored (Fig. 11A). Also, the expression levels of Pim-1 in different clones were examined by performing Western blot analysis (Fig. 11B). In order to determine whether Pim-1 knockdown in an LCL background has some implications in apoptotic cell death, we performed apoptosis assays using stable sh-Ctrl LCL1, sh-Pim-1 LCL1 cells with or without serum starvation. Cells were stained with Propidium iodide for FACS analysis. The results showed substantial increase in apoptotic cell death in stable Pim-1 knockdown LCL1 with serum starvation (Fig. 11C, D).

Programmed cell death or apoptosis is considered as a major regulator of cellular growth control and tissue homeostasis [70]. Previous reports suggested that caspases activation can be triggered through the induction of the extrinsic apoptotic pathway or at the mitochondria by stimulating the intrinsic apoptotic pathway in response with anticancer chemotherapy [71]. In order to determine whether the inhibition of Pim-1 had some effect on apoptotic event in LCLs, we performed Western blot analysis to monitor the levels of PARP-1 cleavage. Our result showed that Pim-1 knock-down EBV transformed cells showed higher signals for the PARP-1 cleavage (Fig. 11E). Moreover, we detected higher expression levels of Caspase-3, Caspase-9, and Apaf-1 in Pim-1 stable knockdown LCL1 in comparison with sh-Ctrl LCL1 cells which indicates that Pim-1 knockdown induced the intrinsic apoptotic pathway in EBV transformed cells (Fig. 11F). We performed cell proliferation assays in the context of Pim-1 knock-down. Interestingly, the result showed that the rate of proliferation of Pim-1 stable knock-down LCL1 cells was lower compared with LCL1 and sh-Ctrl LCL1 cells (Fig. 11G).

### EBNA3C potentiates oncogenic Pim-1 to promote cell proliferation by inhibiting the growth suppressive properties of p21

**Figure 12:**
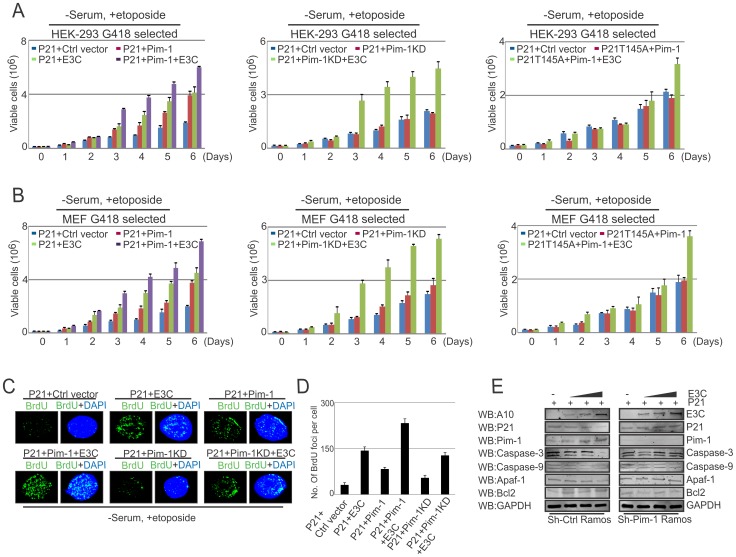
EBNA3C mediated oncogenic Pim-1 activation promotes cell proliferation by impeding the growth suppressive properties of p21. A–B) 10 million HEK-293 and MEF cells were transfected with different combinations of expression plasmids as indicated (A, P21+Ctrl vector; B, P21+Pim-1; C, P21+EBNA3C; D, P21+Pim-1+EBNA3C; E, P21+Pim-1KD; F, P21+Pim-1KD+EBNA3C; G, P21T145A+Pim-1; H, P21T145A+Pim-1+EBNA3C). Transfected cells were selected for 2 weeks with G418 antibiotic. Cells were incubated for 12 hrs in DMEM medium containing 0.1% FBS with 5 µM etoposide. 0.1×10^6^ cells from each set of selected samples were plated and cultured for 6 days. Viable cells were counted at indicated time points by trypan blue dye exclusion technique. B) G418 selected stable cells were harvested, lysed in RIPA buffer and subjected to immunoblot analyses with indicated antibodies. C) 10×10^6^ HEK-293 were transfected with different combinations of expression vectors and transiently transfected cells were subjected to BrdU incorporation assay by immunofluorescence study with etoposide treatment in DMEM containing 0.1% FBS. D) Bar diagram represents number of BrdU foci per cell. The results shown here are representative of three independent experiments. E) 30 million sh-Ctrl and sh-Pim-1 Ramos cells were co-transfected with p21 and increasing amount of EBNA3C expression vectors. Transfected cells were subjected to Western Blot analysis by using indicated antibodies.

As an inhibitor of cyclin-dependent kinases, p21Waf1/Cip1 is required for proper cell-cycle progression [72]. Earlier reports suggested that p21 suppresses tumors by promoting cell cycle arrest in response to various stimuli [61]. In addition, substantial evidence from biochemical and genetic studies shows that p21 acts as a potential effector of multiple tumor suppressor pathways to promote its anti-proliferative activities independent of p53 [61]. Interestingly, several studies demonstrated that ubiquitin-mediated degradation of p21 can also promote cancer cell proliferation [73]. Our results above indicated that p21 is targeted by EBNA3C through Pim-1 dependent degradation. We next attempted to examine whether EBNA3C has a role in modulating p21-mediated inhibition of cell proliferation involving Pim-1 kinase with DNA damage response. HEK-293 and MEF cells were transfected with different combinations of Flag-tagged p21 (wild type and the T145A mutant), Myc-Pim-1 (wild type and the kinase dead mutant), EBNA3C expression vectors. Cell proliferation assays were performed without serum and with etoposide treatment after 2 weeks of G418 antibiotic selection. The results demonstrated that EBNA3C together with wild type Pim-1 effectively reduced the growth suppressive effect of p21. The cell proliferation rate in p21 expressing cells either with EBNA3C or wild type Pim-1 was shown to be enhanced compared to control vector alone. Interestingly, the lower rate of cell proliferation was observed with kinase dead Pim-1 mutant or P21T145A mutant even in the presence of EBNA3C (Fig. 12A, B). To check the expression levels of these proteins, we performed Western blot analysis with these G418 selected cells (Fig. S5A and S5B). Moreover, our immunofluorescence studies for BrdU incorporation with DNA damage showed an increased number of BrdU foci with wild type Pim-1 and p21, co-expressed cells with EBNA3C (Fig. 12C and 12D). To determine the possible contribution of different molecules which are involved with intrinsic apoptotic signaling in the context of Pim-1 mediated p21 downregulation in the presence of EBNA3C, we checked the protein expression profiles of Caspase-3, Caspase-9, Apaf-1, and Bcl2 in sh-Ctrl-vector transfected and Pim-1 stable knockdown EBV negative Ramos cells. These cells were transfected with p21 and an increasing dose of EBNA3C. Our results demonstrated that the expression levels of Caspase-3, Caspase-9, Apaf-1 were unchanged with P21 transfection in Pim-1 knockdown Ramos cells, in the presence of EBNA3C compared with sh-Ctrl-Ramos cells where the expression of these proteins were reduced (Fig. 12E, compare left and right panels). Interestingly, Bcl2 levels were found to be upregulated in sh-Ctrl cells (Fig. 12E). These results support our hypothesis that EBNA3C can potentiate Pim-1 kinase activities for inhibiting cell growth suppressive property of p21 which occurs through the intrinsic apoptotic pathway.

## Discussion

Pim-1 was identified in murine leukaemia virus (MuLV)-induced lymphomas that frequently contains proviral insertions which were associated with the transcriptional activation of the Pim-1 gene frequently associated with enhanced tumorigenesis [74]. Overexpression of Pim kinases have been found in various lymphomas and leukemias [75]. Different reports have suggested a role for Pim-1 kinase in progression of Burkitt's lymphoma [76], primary cutaneous large B-cell lymphoma [77] and prostate cancer [78]. Pim-1 kinase also performs multiple cellular functions related to cell survival, proliferation, differentiation, apoptosis, and progression of tumors [79].

Previous studies showed upregulation of Pim kinases during Epstein-Barr virus infection [45]. Epstein–Barr virus (EBV) was found potentially involved in the pathogenesis of different B-cell lymphoproliferative disease and all three EBV nuclear antigen 3 proteins can manipulate the expression of a wide range of cellular genes and they often act co-operatively to induce epigenetic chromatin modifications [80]. Another report demonstrated that the involvement of EBNA3A and EBNA3C expression with polycomb complexes for the covalent K27me3 modification of histone H3 at the p16INK4A promoter to repress the transcription [81]. Also, BIM expression was regulated in latently infected EBV cells through epigenetic modification and CpG methylation [11]. EBNA3C, regulates transcription of a wide range of viral and cellular genes [14]. EBNA3C was found to be associated with Nm23-H1 to regulate the transcription process of cellular genes which are critically involved in cell migration and invasion [82]. Recent reports also demonstrated that EBNA3C can physically interact and stabilize different host oncoproteins, including c-Myc and IRF4 [20], [51], and has a major role in regulation of the cell cycle regulatory protein complex Cyclin D1/CDK6 to drive B-cell malignancies [22]. Interestingly, some reports showed that Pim-1 levels are tightly controlled at many steps from the transcriptional to translational levels [60]. Our study now demonstrated upregulation of Pim-1 expression at the mRNA and protein levels with EBV infection in primary B-cells as well as EBV positive cancer cell lines. Interestingly, infection with the ΔEBNA3C BAC-GFP-EBV showed a much lower Pim-1 expression at 2 days post-infection. However, the expression patterns remain unchanged at later time points. Our results from the primary infection studies suggested a major contributory role of EBNA3C in inducing Pim-1 expression. We also observed a substantial reduction in Pim-1 expression levels only after siRNA mediated knockdown of EBNA3C but not with the knockdown of EBNA2, EBNA3A and EBNA3B which further confirmed a direct role of EBNA3C in regulating Pim-1 expression levels in EBV-transformed cells. Moreover, our studies showed that EBNA3C has a strong physical association with Pim-1, and that Pim-1 binds to the N-terminal 130–159 residues of EBNA3C. Interestingly, several other studies from our Lab also demonstrated that this region of EBNA3C specifically interacts with different important cellular proteins such as cyclin A, p53, E2F1, c-Myc, IRF4/IRF8 etc [20], [21], [46], [51], [83]. Therefore, this 130–159 aa residues of EBNA-3C have particular significance in deregulating major cellular process in EBV-infected cells. Further detailed investigation is needed to evaluate the functional role of this domain in connection with EBNA3C mediated oncogenesis. Our co-immunoprecipitation experiments in EBNA3C expressing Burkitt's lymphoma cells and EBV transformed Lymphoblastoid cells also demonstrated that Pim-1 forms a strong molecular complex with EBNA3C in infected cells.

Previous studies suggested that nuclear localization of Pim-1 is essential for the regulation of its cellular substrates as well as additional biological activities of this kinase [76]. Importantly, our co-localization studies showed that in the absence of EBNA3C, localization of Pim-1 was mostly in the cytoplasm and predominantly in the nucleus in the presence of EBNA3C. Our immunofluorescence assay therefore revealed a strong co-localization with Pim-1 and EBNA3C in the nucleus. Previously it was shown that the Hsp90 protein is responsible for correct folding and stabilization of Pim-1 [84]. Further, studies from our group demonstrated an important role of EBNA3C in stabilizing different oncoproteins such as Gemin3, Cyclin D1, and IRF4 [19], [22], [51] to deregulate normal cellular functions which can drive development of neoplastic events. Our study clearly demonstrated that Pim-1 protein stabilization by EBNA3C can result in increased levels of Pim-1 in EBV infected cells. Additionally, the stability of the Pim-1 kinase is largely regulated by the ubiquitin/proteasome pathway [85]. Several reports suggested the important role of EBNA3C for deregulating the functions of different cellular proteins by manipulation of ubiquitin/proteasome pathways [86]. Our Lab previously demonstrated the interaction between EBNA3C with SCF^Skp2^ E3 ligase complex [87]. Also, the N-terminal domain of EBNA3C physically associated with the acidic domain of Mdm2 which is a known E3 ubiquitin-protein ligase [50]. Other studies also suggested that EBNA3C associates with the α-subunit of the 20S proteasome and is degraded in-vitro by purified 20S proteasomes [88]. EBNA3C was found to facilitate the degradation of E2F1 by targeting ubiquitin-proteasome pathways [46]. Recently, we have shown that EBNA3C deregulates total H2AX levels through involvement of the ubiquitin/proteasome degradation pathway [89]. Interestingly, other reports suggested the potential involvement of Pim-1 with ubiquitin/proteasome pathways as enhanced expression of Pim-1 increases the level of SCF^Skp2^ ubiquitin ligase through the direct binding and phosphorylation of multiple sites on this protein [90]. A previous study showed the role of heat shock proteins and the ubiquitin-proteasome pathway for regulating the stability of Pim-1 kinase [85]. Our poly-ubiquitination experiments clearly suggested that Pim-1 poly-ubiquitination was significantly inhibited by EBNA3C and so resulted in increased Pim-1 levels. Since enhanced levels of Pim-1 is linked to different hematological or non-hematological malignancies, it reveals the intricate mechanisms that are linked to ubiquitin-proteasome-mediated degradation of Pim-1 and is important for designing therapeutic interventions. Additionally, this approach could enhance new therapeutic avenues by targeting Pim-1 kinase and so enhance the efficiency of conventional therapeutic strategies against EBV mediated oncogenesis.

Being a potent serine/threonine kinase, Pim-1 plays important roles in a number of cellular events. Most notably, Pim-1 can synergize with c-Myc to drive the rapid progression of B-cell lymphomas [91]. This synergism is likely to originate from the anti-apoptotic activity promoted by Pim-1 [92]. Among other Pim-1 substrates, the Cyclin-dependent kinase inhibitor 1 or p21 is important in the context of viral pathogenesis. Interestingly, p21 stability has been exploited by different tumor viruses. A number of viral proteins can affect the post-transcriptional regulation of p21, thereby affecting cellular proliferation. The human papilloma virus E6 proteins can downregulate p21 independently of p53 [93]. Also, the hepatitis C virus and K-cyclin encoded by the human herpesvirus 8 stimulates p21 phosphorylation at the Ser130 residue by CDK6 without affecting its stability [94]. These findings further establish that targeting p21 is likely to be a common strategy for viruses to regulate cell cycle progression and apoptosis. Previous reports also showed that EBV acts downstream of the p53 and appears to prevent the inactivation of cyclin-dependent kinase CDK2 by p21^WAF1/CIP1^ by targeting p21 for degradation by the proteasome pathway [95]. Basically, participation of p21 in multiple cellular functions emphasizes its importance and that its precise regulation is crucial for maintenance of the normal cellular function. Importantly, its phosphorylation and interaction with other cellular proteins are crucial to p21 stability at the post-translational level. Previous reports suggested that the Thr145 residue of p21 is preferentially phosphorylated by Pim-1 [36]. Our current study clearly demonstrated a role for EBNA3C in enhancing Pim-1 kinase activity to phosphorylate p21. We also observed that Pim-1 was not able to phosphorylate mutant p21 (T145A) even in the presence of EBNA3C. Interestingly, we identified a molecular association between Pim-1 and p21, with EBNA3C and our competitive binding assay demonstrated that increasing doses of EBNA3C resulted in reduced association between Pim-1 and p21, causing in destabilization of p21 by enhancing its proteasome-mediated degradation independent of etoposide induced DNA damage response.

Several reports indicated that Pim-1 expression is associated with cell proliferation and survival [96]. Pim-1 also induces anti-cancer drug resistance by inhibiting the intrinsic mitochondrial apoptosis pathway [97]. In our studies, siRNA mediated knock down of Pim-1 showed reduced proliferation of EBV transformed cells. Moreover, Pim-1 silencing potentially activated the intrinsic apoptotic signaling in EBV transformed cells. Recent studies showed that upregulation of p21 activated the intrinsic apoptotic pathway [98]. Our results also support this finding showing that EBNA3C induced Pim-1 mediated downregulation of p21 which is also related to the inhibition of intrinsic apoptotic pathway in EBV transformed cells. Recent evidence suggested a role for the RNF126 E3 ubiquitin ligase in promoting cancer cell proliferation by p21 degradation [73]. Our results strongly suggested an important role for EBNA3C to effectively inhibit the growth suppressive effects of p21 in the presence of Pim-1. Interestingly, we observed a lower rate of cell proliferation with the kinase dead Pim-1 mutant or P21T145A mutant even in the presence of EBNA3C. This supports a role for Pim-1-mediated phosphorylation of the Thr145 residue of p21 in cell proliferation.

**Figure 13:**
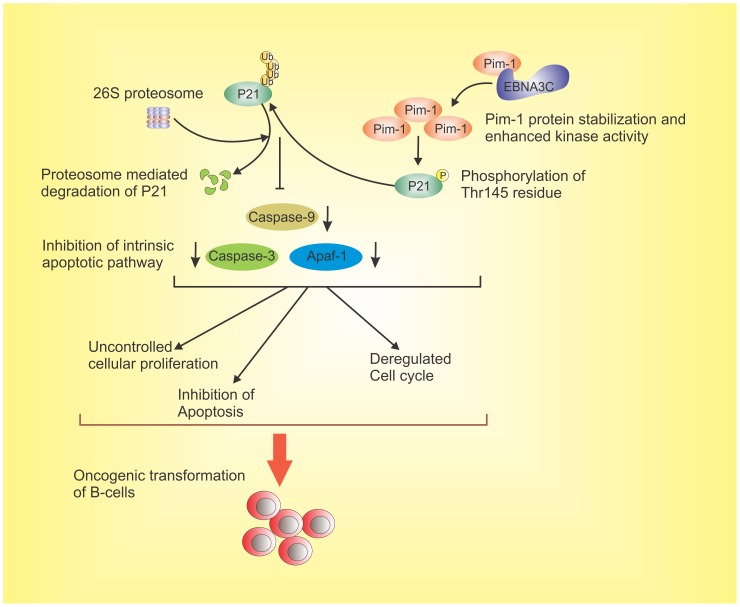
The schematic diagram illustrates the potential contribution of EBNA3C to oncogenic transformation of B-cells through stabilization of Pim-1 and proteasome mediated degradation of p21 which results in inhibition of the intrinsic apoptotic pathway. EBNA3C interacts with oncogenic serine/threonine Pim-1 kinase. This molecular association stabilizes Pim-1 by inhibiting its Poly-ubiquitination. EBNA3C also promotes Pim-1 mediated p21 degradation. EBNA3C contributes to B-cell proliferation by activating oncogenic Pim-1 which leads to inhibition of the growth suppressive property of p21 as well as impeding intrinsic apoptosis signaling. This molecular strategy for oncogenic Pim-1 kinase targeted by EBNA3C for inhibition of p21 function, and it can be a potential therapeutic target for EBV-associated malignancies.

In summary, our current work demonstrated an important molecular mechanism which revealed a direct role for the EBV latent antigen 3C in enhancing expression of the oncoprotein Pim-1 in EBV transformed B-cells as well as in EBV-infected PBMCs. We also showed the physical interaction between EBNA3C and Pim-1 and further mapped the binding to the Amino-terminal domain of EBNA3C. Moreover, our study demonstrated that EBNA3C mediated stabilization of Pim-1 through abrogation of the proteasome/ubiquitin pathway. EBNA3C also facilitated the nuclear export of Pim-1 and promoted EBV transformed cell proliferation by altering Pim-1-mediated regulation of the cell-cycle inhibitor p21/WAF1 activity. Our study now demonstrated that EBNA3C directly contributes to Pim-1 mediated phosphorylation of p21 which facilitates its proteosomal degradation. In addition, significant reduction of EBV transformed cell proliferation as well as a substantial induction of apoptotic cell death was also observed upon stable knockdown of Pim-1. Our study now provides a novel insight into the precise role of oncogenic Pim-1 in EBV-mediated oncogenesis (Fig. 13). Moreover, siRNA mediated knockdown of Pim-1 triggers the intrinsic apoptotic signaling pathway in LCLs and repressed proteasome-mediated degradation of p21. Pim-1 knockdown further demonstrated a vital role in EBV-mediated proliferation of B-cells by impeding the process of apoptosis. Our findings thus contribute to a more indepth understanding of the role of EBNA3C expressed in EBV-infected B-cells and its interaction with the critical cellular kinase which leads to EBV induced B-cell transformation.

## Materials and Methods

### Ethics statement

PBMC were obtained from University of Pennsylvania Human Immunology Core (HIC) and donated by the healthy donors. This study was approved by University of Pennsylvania Human Immunology Core (HIC) which maintains University of Pennsylvania IRB protocol. In this IRB approved protocol the declarations of Helsinki protocols were followed and each donor gave written, informed consent. There is no link between donors and their information with this study.

### Plasmids, antibodies and cell lines

Full length and truncated mutants of GST, Myc, Flag, and GFP tagged EBNA3C expression vectors were described previously [22], [51]. Myc-tagged EBNA3C with C143N point mutation was generated by using standard PCR primer mutagenesis method [50]. Constructs for Myc-tagged Pim-1, kinase dead (KD) version of Pim-1 as Pim-1 K67M (mutated at the ATP binding Pocket), pGEX2T-Pim-1 were mentioned previously [43]. Wild type pGEX-P21 construct was generated by using Flag-P21 construct as template. The PCR amplified insert was subjected for EcoRI/NotI restriction enzyme digestion and ligated into pGEX2T vector. pGEX-P21T145A and Flag-P21T145A constructs were cloned by using PBK/CMV/LacZ P21T145A (kindly provided by Dr. Nancy Magnuson) as a template for the PCR amplification. pCDNA3-HA-Ub construct was kindly provided by George Mosialos (Aristotle University of Thessaloniki, Greece) and pGIPZ was used as the sh-RNA vector described previously [51]. Constructs used for lentiviral packaging were previously described [99].

Antibodies of Pim-1 (E-16), Ub (FL-76), PARP-1 (F-2), and GFP (I-16), Caspase-3 (E-8), Caspase-9 p10 (H-83), Apaf-1 (H-324), Bcl2 (C-2) were purchased from Santa Cruz Biotechnology, Inc (Santa Cruz, CA). P21 (ab7960) antibody was purchased from Abcam (Cambridge, MA). GAPDH antibody was procured from US-Biological Corp. (Swampscott, MA). Flag (M2)-epitope, anti-mouse antibody was purchased from Sigma-Aldrich Corp. (St. Louis, MO). Hybridomas for mouse anti-Myc (9E10), anti-Hemaggutinin (12CA5), A10 were previously described [99].

HEK-293 (human embryonic kidney cell line) was kindly provided by Jon Aster (Brigham and Woman's Hospital, Boston, MA, USA). HEK-293, and MEF cells were grown in Dulbeccoo's modified Eagle's medium (DMEM). EBV negative Burkitt's lymphoma cells BJAB, DG75, Ramos were kindly provided by Elliot Kieff (Harvard Medical School, Boston, MA). BJAB stably expressing EBNA3C (BJAB7, BJAB10) were previously described [76]. EBV transformed lymphoblastoid cell lines (LCL1, LCL2) were maintained in RPMI 1640 media

### Transfections

Transfection in HEK-293, MEF and B-cells were performed by electroporation system with Bio-Rad Gene Pulser II electroporator. Cells were electroporated at 210 V and 975 µF (for HEK-293, MEF cells) or 220 V and 975 µF (for DG75, Ramos, LCL1 cells).

### Infection of PBMCs with BAC-GFP wild type EBV and ΔE3C-EBV

PBMCs (Peripheral blood mononuclear cells) were obtained from healthy donors from University of Pennsylvania Immunology Core as mentioned previously [100]. As defined earlier [46], 10 million PBMCs were mixed with wild type and EBNA3C knockout mutant (BAC-GFP-ΔE3C-EBV) virus supernatant in 1 ml of RPMI 1640 media containing 10% FBS for 4 hrs at 37°C and 5% co_2_ environment. Next, cells were centrifuged at 500×g for 5 minutes and pelleted cells were again re-suspended in 2 ml of complete medium. EBV-GFP expression was checked by fluorescence microscopy and used to evaluate the infection. Infected cells were harvested at specific time intervals to determine the Pim-1 protein and mRNA levels.

### GST-pulldown assay

For GST pull-down assays, cell lysates from BJAB, BJAB7, BJAB10, LCL1, LCL2 cells were incubated with bacterially purified control GST protein and GST fusion proteins. Protein samples were washed using Binding Buffer (1× PBS, 0.1% NP-40, 0.5 mM DTT, 10% glycerol, with protease inhibitors) and resolved by 10% SDS-PAGE. A10 antibody was used for Western blot analysis.

### Immunoprecipitation and western blot analysis

Cells were harvested and washed with 1× Phosphate Buffered Saline (PBS). For the preparation of cell lysates, RIPA buffer (0.5% NP-40, 10 mM Tris pH 7.5, 2 mM EDTA, 150 mM NaCl, supplemented with 1 mM PMSF, and protease inhibitors) was used. Cell lysates were then pre-cleared with normal mouse/rabbit serum rotating with 30 µl of Protein-A and Protein-G (1∶1 mixture)-conjugated Sepharose beads for 1 hr at 4°C. 5% of the protein lysate was saved as input sample. Appropriate antibody (1 µg/ml) was used to capture the specific protein of interest by rotating the sample overnight at 4°C. The immune-precipitated samples were washed with RIPA buffer. Protein samples were boiled in laemmli buffer [101], resolved by SDS-PAGE and Western blotting was performed. The membranes were probed with appropriate antibodies and scanned using the Odyssey imager (LiCor Inc., Lincoln, NE).

### Real-time PCR analysis

Ice-cold PBS was used to wash the cells prior to RNA isolation. Trizol reagent (Invitrogen, Inc., Carlsbad, CA) was used for RNA extraction according to manufacturer's protocol. Next, Superscript II reverse transcriptase kit (Invitrogen, Inc., Carlsbad, CA) was used for cDNA preparation according to the manufacturer's instructions. The primers for Pim-1, EBNA3C, EBNA2, EBNA3A, EBNA3B were 5′-CGAGCATGACGAAGAGATCAT-3′ and 5′-TCGAAGGTTGGCCTATCTGA-3′ [102], 5′-AGAAGGGGAGCGTGTGTTGT-3′ and 5′-GGCTCGTTTTTGACGTCGGC-3′, 5′- GAACTTCAACCCACACCATC-3′ and 5′- CGTGGTTCTGGACTATCTGG-3′, 5′- GGTGAAACGCGAGAAGAAAG-3′ and 5′- CTCTCATCAGCAGCAACCTG-3′, 5′- AGAAGAGGCCCTTGTGTCTT-3′ and 5′- GGATTTCAAGAGGGTCAGGT-3′ respectively. GAPDH primers were used as 5′-TGCACCACCAACTGCTTAG-3′ and 5′-GATGCAGGGATGATGTTC-3′ [22]. SYBER green Real-time master mix (MJ Research Inc., Waltham, MA) was used for quantitative real-time PCR analysis. To check the specificity of the products a melting curve analysis was performed and the relative quantitation values were calculated by threshold cycle method. Triplicate sets were used to examine each sample.

### Immunofluorescence assay

300 thousand HEK-293 cells were transfected with different expression plasmids by Lipofectamine 2000 transfection reagents (Invitrogen, Carlsbad, CA). Cells were fixed with 3% paraformaldehyde (PFA) with 0.1% Triton X-100 and 1% BSA was used for blocking purpose. Myc-tagged Pim-1 was detected by using anti-Myc (9E10) antibody and the expression of GFP-tagged EBNA3C was detected by GFP-fluorescence. BJAB, BJAB10, and LCL1 cells were semi-air-dried on slides and fixed as mentioned above. Specific antibodies were used to check endogenous expressions of EBNA3C and Pim-1. Nuclear staining was performed by using DAPI (4′,6′,-diamidino-2-phenylindole; Pierce, Rockford, IL). After secondary antibody and DAPI incubation, cells were washed in 1× PBS and mounted with antifade mounting medium. The images were taken by Fluoview FV300 confocal microscope and FLUOVIEW software (Olympus Inc., Melville, NY) was used for image analysis.

### Nuclear and cytosolic fractionation assay

HEK-293 cells were transfected with combinations of expression vectors. After 36 hrs of post-transfection, cells were washed with PBS and re-suspended into hypotonic buffer [5 mM Pipes (KOH) pH 8.0, 85 mM KCl, 0.5% NP-40 supplemented with protease inhibitors). Cells were incubated on ice, and Dounce homogenizer was used to homogenize the cells with 20 strokes. Nuclei were pelleted down (2300×g for 5 min) and the cytosolic fraction was collected. Nuclear pellets were washed again with PBS, re-suspended in nuclear lysis buffer (50 mM Tris, pH 8.0, 2 mM EDTA, 150 mM NaCl, 1% NP-40, and protease inhibitors) and lysed by vortexing intermittently for 1 h. The soluble nuclear fraction was separated by centrifugation at 21,000×g for 10 min. To determine the efficiency of nuclear and cytoplasmic fractionation, Western blot analysis was done against the nuclear transcription factor SP1 and cytoplasmic protein GAPDH using specific antibodies.

### Poly-ubiquitination assay

Expression vectors were transfected by electroporation in HEK-293 cells. Transfected cells were incubated for 36 hrs in fresh DMEM and treated with 20 µM MG132 (Enzo Life Sciences International, Inc., Plymouth Meeting, PA) for another 6 hrs. Protein samples were immunoprecipitated with appropriate antibodies and resolved by SDS-PAGE. The level of ubiquitination was detected by HA-specific antibody (12CA5).

### In-vitro kinase assay

Myc-tagged-Pim-1 (wild type or kinase dead mutant) (5 mg), Flag-EBNA3C (5 mg) expression constructs were transfected in HEK-293 cells. After 36 hrs of post-transfection, cell lysates were prepared and protein complexes were immunoprecipitated (IP) by using 9E10 ascites fluid. IP complexes were then washed with buffer A (containing 25 mM Tris [pH 7.5], 70 mM NaCl, 10 mM MgCl2, 1 mM EGTA, 1 mM DTT, with protease and phosphatase inhibitors) and incubated in 30 ml of kinase buffer B (containing buffer A plus 10 mM cold ATP, and 0.2 mCi of [c-32P]-ATP/ml) supplemented with bacterially purified GST-P21 (wild type or T145A mutant) for 30 min at 30°C. 2× laemmli buffer was added to stop the reaction and heated at 95°C for 10 min. 10% SDS-PAGE was used for resolving the labeled proteins. Quantitation of the band was performed by using Image Quant software (GE Healthcare Biosciences, Pittsburgh, PA).

### Stability assay

Transient transfection was performed in 10 million HEK-293 cells using electroporation system with combinations of plasmids as mentioned in the text. After 36 hours transfection, transfected cells as well as B-cells were treated with 40 µg/ml cyclohexamide in specific time periods with DNA damage response and cell lysates were prepared with RIPA buffer. Protein samples were subjected to Western blot analysis. Odyssey 3.0 software was used to quantify the band intensities.

### Production of lentivirus and si-RNA mediated gene silencing by transduction of EBV transformed cells

Short-hairpin (sh) oligonucleotides directed against EBNA3C were described previously [22]. The Pim-1 target sequence 5′-GUGUACUUUAGGCAAAGGG-3′ was described previously [43]. sh-oligonucleotides used for EBNA2, EBNA3A and EBNA3B knockdown were 5′- TCGAGTTGTTGACACGGATAGTCTTTCAAGAGAAGACTATCCGTGTCAACAATTTTTTA-3′ and 5′- CGCGTAAAAAATTGTTGACACGGATAGTCTTCTCTTGAAAGACTATCCGTGTCAACAAC-3′, 5′- TCGAGGAACACTTCTTCAAGCGATTTCAAGAGAATCGCTTGAAGAAGTGTTCTTTTTTA-3′ and 5′- CGCGTAAAAAAGAACACTTCTTCAAGCGATTCTCTTGAAATCGCTTGAAGAAGTGTTCC-3′, 5′- TCGAGTTGATTGTCATTGGTTTCATTCAAGAGATGAAACCAATGACAATCAATTTTTTA-3′ and 5′- CGCGTAAAAAATTGATTGTCATTGGTTTCATCTCTTGAATGAAACCAATGACAATCAAC-3′ respectively. EBNA3C and Pim-1 specific oligonucleotides were cloned into pGIPZ vector at XhoI and MluI restriction sites. Control shRNA sequence (Dharmacon Research, Chicago, IL) was used as 5′-TCTCGCTTGGGCGAGAGTAAG-3′ which lacks complementary sequences in the human genome, also cloned in pGIPZ vector. Lentivirus production and transduction of EBV-transformed LCL1 were described previously [51].

### Colony formation assay

10 million Human kidney embryonic cells were subjected to transient transfection with Ctrl-vector, Myc-Pim-1, and Flag-tagged-EBNA3C by electroporation system. Transfected cells were allowed to grow in DMEM containing G418 as 1 mg/ml concentration. After selecting the cells up to 2-weeks, selected cells were fixed with 4% formaldehyde and stained with 0.1% crystal violet solution (Sigma-Aldrich Corp., St. Louis, MO). The area of the colonies was calculated by using Image J software (Adobe Inc., San Jose, CA). The data shown here are average of three independent experiments.

### Cell proliferation assay

HEK-293, MEF cells were transfected with different combinations of expression vectors by electroporation as described in the text. Transfected cells were grown in DMEM and were selected with 1000 µg/ml G418 antibiotic for 2-weeks. After selection, Cells were incubated without serum and with etoposide (MP Biomedicals, LLC) treatment for 12 hrs. Cell lysates were prepared by RIPA buffer and protein expression was examined by Western blotting. From each transfected and selected set, 0.1×10^6^ cells were plated and allowed to grow them for 6 days. Also, LCL1, sh-Ctrl LCL1 and sh-Pim-1 LCL1 cells were plated and grown in RPMI media. Counting of viable cells at specific time points was performed by using Trypan Blue dye exclusion method. All experiments were performed in triplicates.

### BrdU incorporation assay

HEK-293 cells were transfected with specific plasmid vectors as indicated in the text. After 36 hrs of post-transfection, BrdU was added and incubated cells for 2 hours in the presence of DNA damaging agents. Cells were fixed with 4% paraformaldehyde (PFA) for 15 min in room temperature. Cells were washed with PBS. 2 M HCl was then added and incubated for 20 min at room temperature. Next, 0.1 M sodium borate (Na_2_B_4_O_7_) pH 8.5 was added and incubated for 2 min at room temperature. Cells were washed with PBS and incubated with 0.2% Triton X100, 3% BSA in 1×PBS for 5 min at room temperature. Cells were washed three times with PBS/BSA for 10 min each. Cells were incubated with anti BrdU antibody in PBS/BSA solution. After washing three times with PBS/BSA solution, cells were incubated for 1 hr with secondary antibody. DAPI was added at the final washing steps to stain DNA. The images were observed by Fluoview FV300 confocal microscope.

### Statistical analysis

Data represented here are as the mean values with standard errors of means (SEM). The significance of differences in the mean values was calculated by performing 2-tailed student's t-test. P-value of <0.05 was considered here as statistically significant.

### Accession numbers

Homo sapiens pim-1- GenBank: M16750.1, Homo sapiens cyclin-dependent kinase inhibitor 1A (p21, Cip1)- GenBank: BC001935.1, Epstein-Barr virus (EBV) genome, strain B95-8- GenBank: V01555.2, human Pim-1 protein- UniProtKB/Swiss-Prot: P11309, human P21 protein- UniProtKB/Swiss-Prot: P38936, EBNA3C protein- UniProtKB/Swiss-Prot: P03204.1.

## Supporting Information

Figure S1 **EBNA3C induces Pim-1 upregulation.** A) 10 million LCL1 cells were transiently transfected with EBNA2, EBNA3A, EBNA3B and EBNA3C knockdown constructs and 36 hrs of post-transfection, total RNA was isolated from the cells and subjected to cDNA conversion. The *Pim-1* transcript level was checked by RT-PCR. The P-values of the mean differences for sh-EBNA2 LCL1, sh-EBNA3A LCL1, sh-EBNA3B LCL1 compared with sh-Ctrl LCL1 for *Pim-1* transcripts are 0.0941, 0.1567, 0.0728, 0.0075 respectively. The error bars indicate standard deviations from three independent experiments. p-value of <0.05 was considered here as statistically significant. B) The knockdown efficiency of EBNA2, EBNA3A, EBNA3B, and EBNA3C was shown by agarose gel after RT-PCR analysis.
(TIF)

Figure S2 **Expression levels of other EBNAs are unaffected by EBNA3C knockdown.** 50 million sh-Ctrl and sh-E3C LCL1 cells were harvested and cell lysates were prepared by RIPA buffer. Protein samples were subjected to Western blot analysis by using human polyclonal serum capable of detecting EBNA proteins expressed by EBV during latent infection. GAPDH was shown as internal loading control. Pim-1 expression level was substantially reduced upon EBNA3C knockdown in LCL1 cells. Expression levels of EBNA1, EBNA2, EBNA3A, EBNA3B, EBNA-LP were not affected with EBNA3C knockdown.
(TIF)

Figure S3 **EBNA3C shows physical association with p21.** A) HEK-293 cells were transfected with Flag-P21 and Myc-EBNA3C expression vector. Immunoprecipitation was performed with anti-Flag antibody. The results showed direct interaction with p21 and EBNA3C by the co-immunoprecipitation experiments. B) p21 protein level was examined by expressing Flag-p21, Myc-Pim-1, and increasing amount of EBNA3C in HEK-293 cells. The results indicated reduced level of p21 with dose dependent increase of EBNA3C in presence of Pim-1.
(TIF)

Figure S4 **Pim-1 accelerates cell proliferation in the presence of EBNA3C.** A) HEK-293 cells were transfected with combinations of control vector, Flag-tagged EBNA3C, Myc-Pim-1 expression vectors, and Myc-Pim-1 with Flag-EBNA3C. Colony formation assays were performed after G418 antibiotic selection for 2 weeks. Here, our results showed a substantial increase in colony numbers with EBNA3C and Pim-1 co-transfection. B) The colony numbers of different transfected sets were represented in bar diagram. The data represented here as the average of three independent experiments. C) The rate of cell proliferation was determined by cell counting using Trypan blue dye exclusion technique for 6 days.
(TIF)

Figure S5 **EBNA3C mediated potentiation of Pim-1 leads to inhibition of p21.** A–B) HEK-293 and MEF cells were transfected with different combinations of Flag-tagged p21 (wild type and the T145A mutant), Myc-Pim-1 (wild type and the kinase dead mutant), EBNA3C expression vectors. The expression levels of these proteins were analyzed by Western blots with indicated antibodies in these G418 selected cells.
(TIF)
