# Supplementary figures and images for: EBNA3C Augments Pim-1 Mediated Phosphorylation and Degradation of p21 to Promote B-Cell Proliferation
Source: PLoS Pathog. 2014 Aug 14;10(8):e1004304. doi: 10.1371/journal.ppat.1004304 (PMC4133388; doi:10.1371/journal.ppat.1004304)

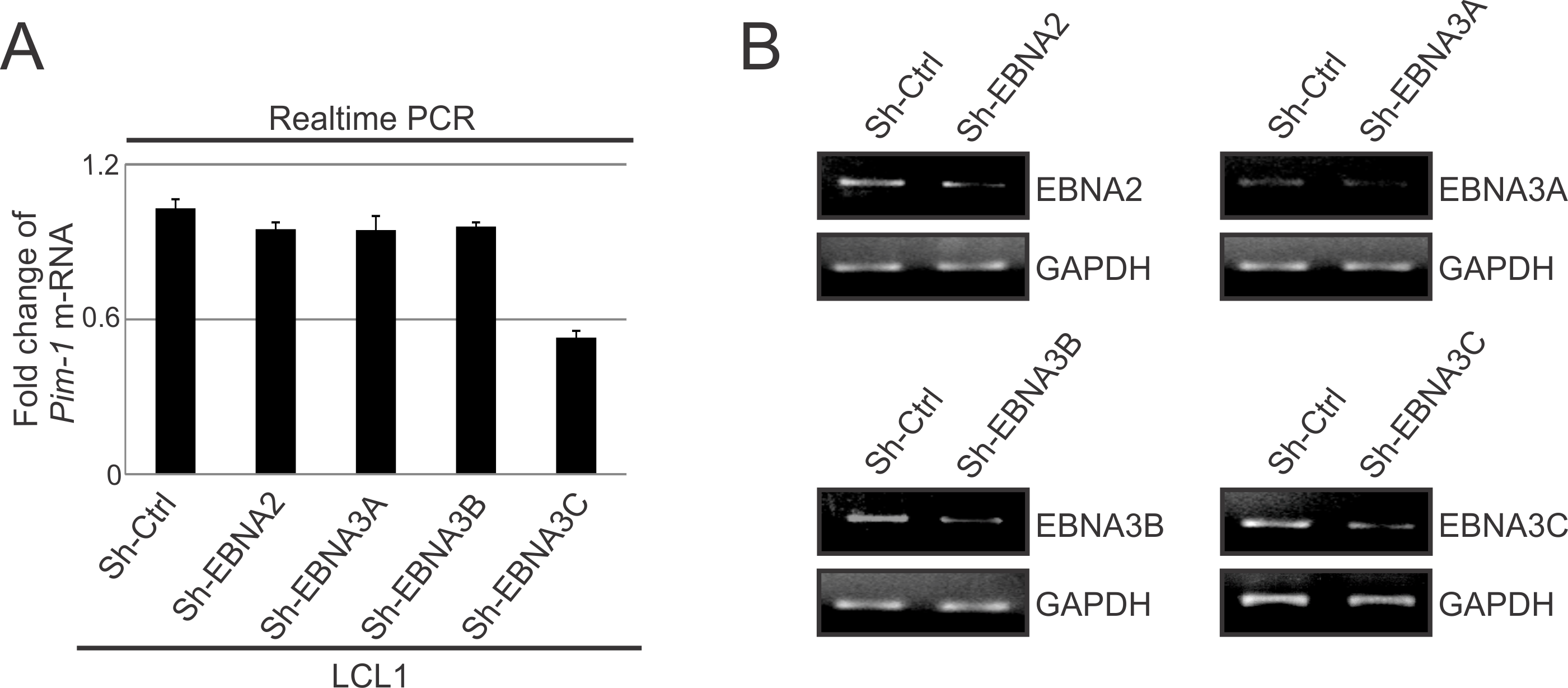

Supplement: Figure S1 — EBNA3C induces Pim-1 upregulation. A) 10 million LCL1 cells were transiently transfected with EBNA2, EBNA3A, EBNA3B and EBNA3C knockdown constructs and 36 hrs of post-transfection, total RNA was isolated from the cells and subjected to cDNA conversion. The Pim-1 transcript level was checked by RT-PCR. The P-values of the mean differences for sh-EBNA2 LCL1, sh-EBNA3A LCL1, sh-EBNA3B LCL1 compared with sh-Ctrl LCL1 for Pim-1 transcripts are 0.0941, 0.1567, 0.0728, 0.0075 respectively. The error bars indicate standard deviations from three independent experiments. p-value of <0.05 was considered here as statistically significant. B) The knockdown efficiency of EBNA2, EBNA3A, EBNA3B, and EBNA3C was shown by agarose gel after RT-PCR analysis. (TIF) [file ppat.1004304.s001.tif]

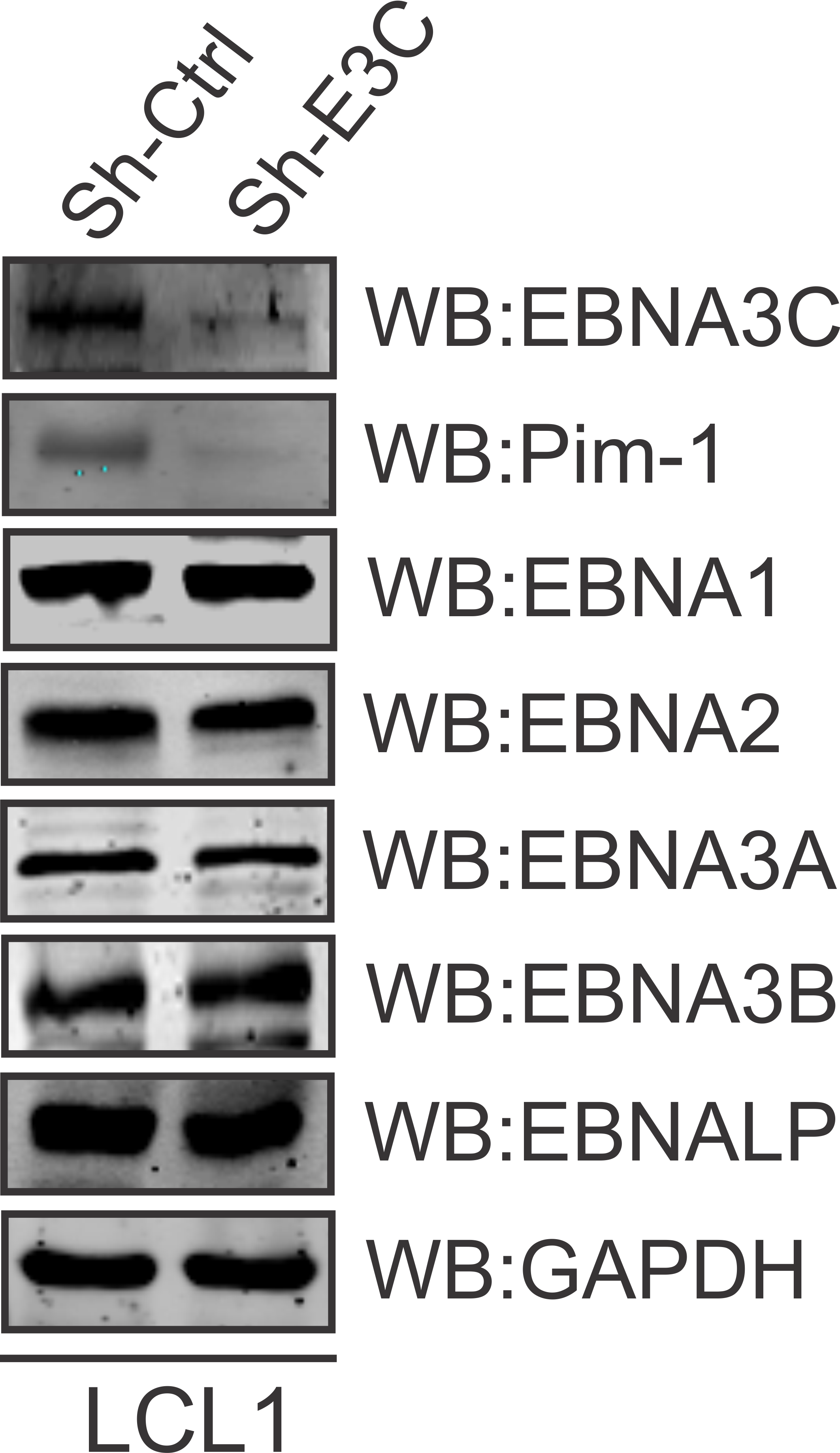

Supplement: Figure S2 — Expression levels of other EBNAs are unaffected by EBNA3C knockdown. 50 million sh-Ctrl and sh-E3C LCL1 cells were harvested and cell lysates were prepared by RIPA buffer. Protein samples were subjected to Western blot analysis by using human polyclonal serum capable of detecting EBNA proteins expressed by EBV during latent infection. GAPDH was shown as internal loading control. Pim-1 expression level was substantially reduced upon EBNA3C knockdown in LCL1 cells. Expression levels of EBNA1, EBNA2, EBNA3A, EBNA3B, EBNA-LP were not affected with EBNA3C knockdown. (TIF) [file ppat.1004304.s002.tif]

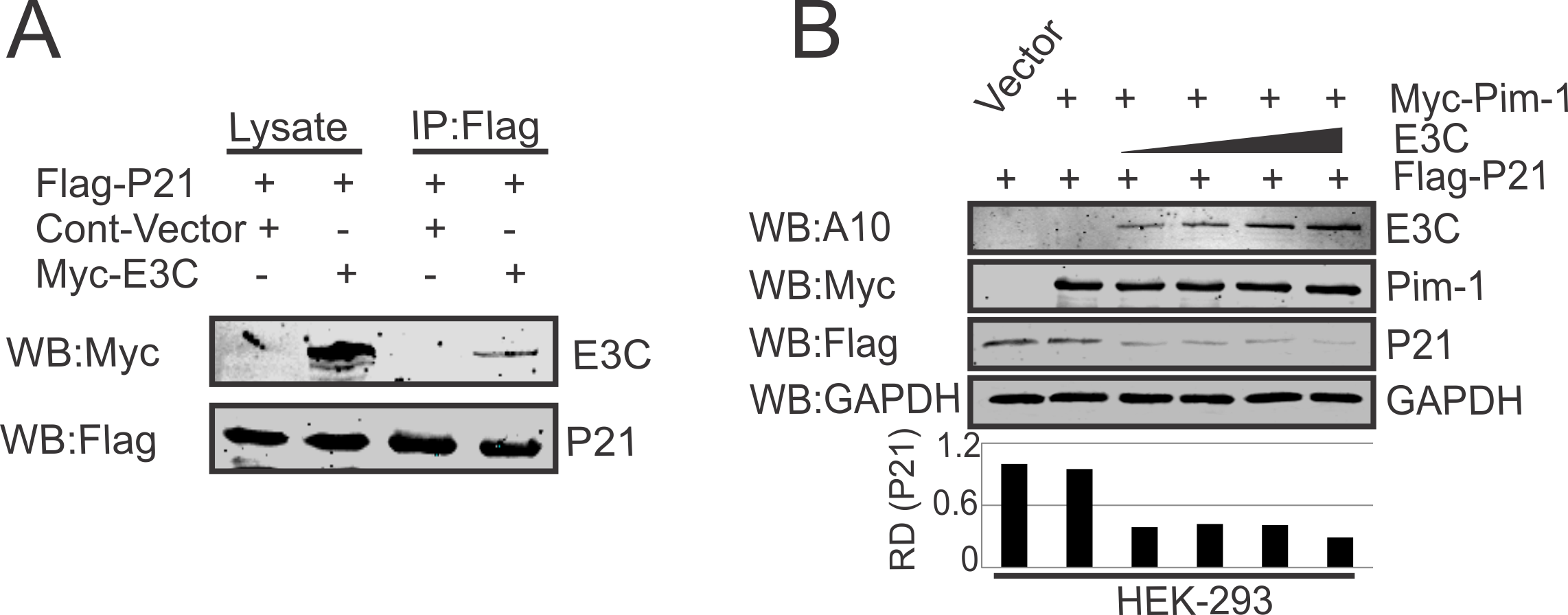

Supplement: Figure S3 — EBNA3C shows physical association with p21. A) HEK-293 cells were transfected with Flag-P21 and Myc-EBNA3C expression vector. Immunoprecipitation was performed with anti-Flag antibody. The results showed direct interaction with p21 and EBNA3C by the co-immunoprecipitation experiments. B) p21 protein level was examined by expressing Flag-p21, Myc-Pim-1, and increasing amount of EBNA3C in HEK-293 cells. The results indicated reduced level of p21 with dose dependent increase of EBNA3C in presence of Pim-1. (TIF) [file ppat.1004304.s003.tif]

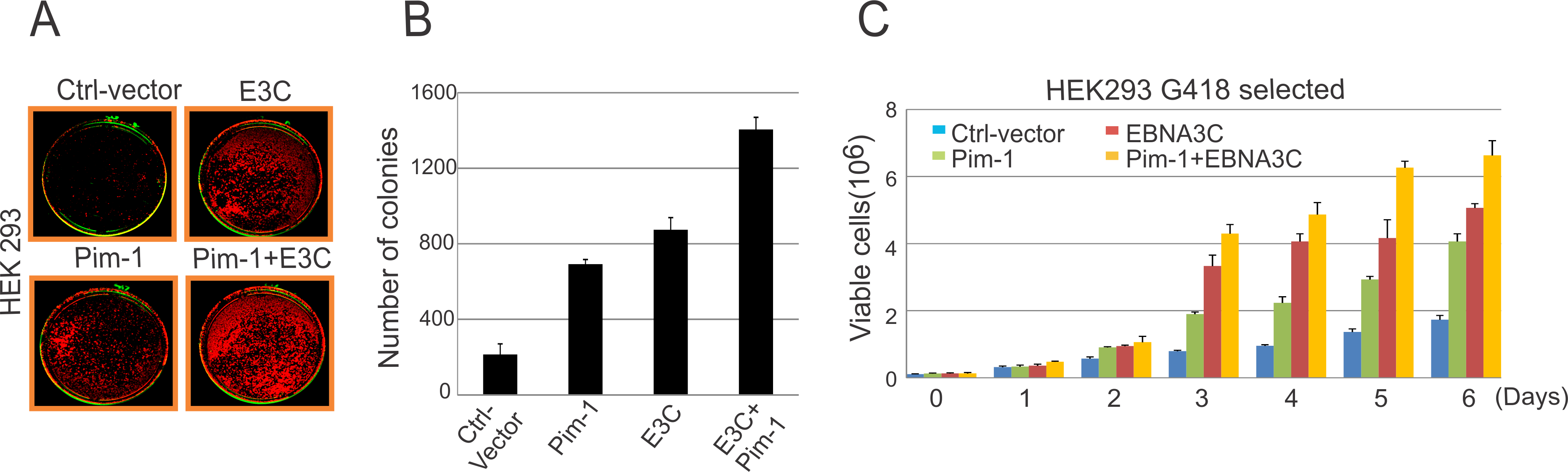

Supplement: Figure S4 — Pim-1 accelerates cell proliferation in the presence of EBNA3C. A) HEK-293 cells were transfected with combinations of control vector, Flag-tagged EBNA3C, Myc-Pim-1 expression vectors, and Myc-Pim-1 with Flag-EBNA3C. Colony formation assays were performed after G418 antibiotic selection for 2 weeks. Here, our results showed a substantial increase in colony numbers with EBNA3C and Pim-1 co-transfection. B) The colony numbers of different transfected sets were represented in bar diagram. The data represented here as the average of three independent experiments. C) The rate of cell proliferation was determined by cell counting using Trypan blue dye exclusion technique for 6 days. (TIF) [file ppat.1004304.s004.tif]

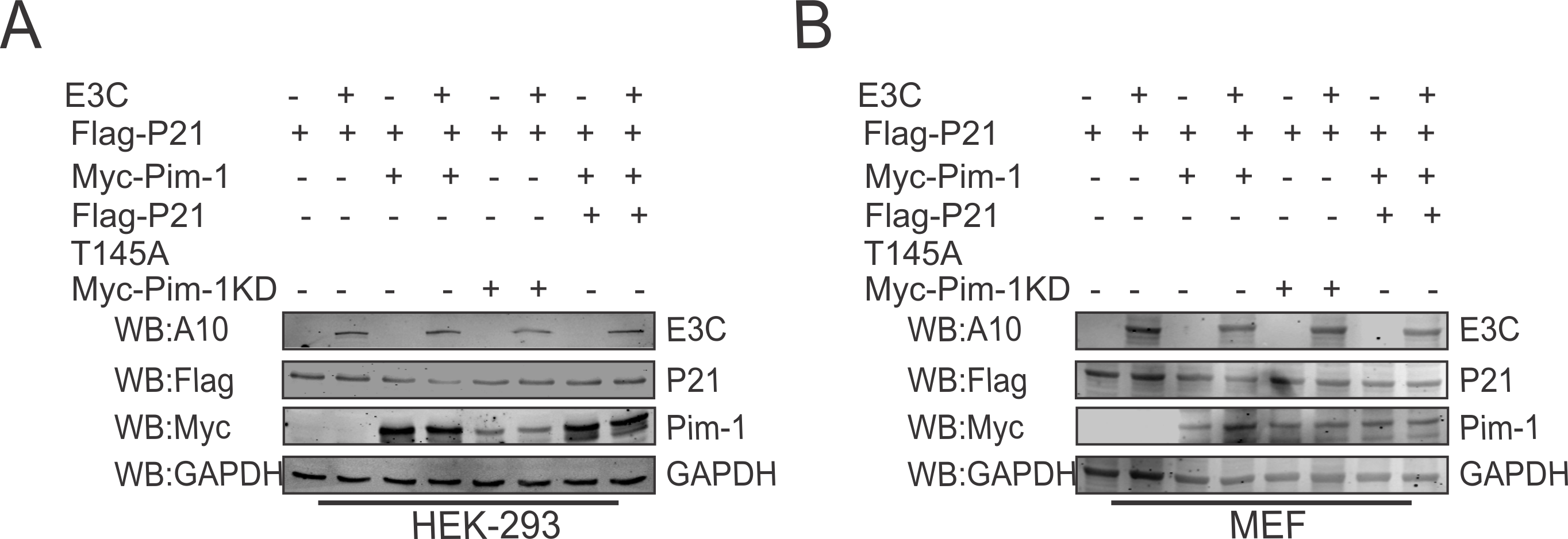

Supplement: Figure S5 — EBNA3C mediated potentiation of Pim-1 leads to inhibition of p21. A–B) HEK-293 and MEF cells were transfected with different combinations of Flag-tagged p21 (wild type and the T145A mutant), Myc-Pim-1 (wild type and the kinase dead mutant), EBNA3C expression vectors. The expression levels of these proteins were analyzed by Western blots with indicated antibodies in these G418 selected cells. (TIF) [file ppat.1004304.s005.tif]
